# Differences in ill health and in socioeconomic inequalities in health by ethnic groups: a cross-sectional study using 2011 Scottish census

**DOI:** 10.1080/13557858.2019.1643009

**Published:** 2019-07-17

**Authors:** Mirjam Allik, Denise Brown, Ruth Dundas, Alastair H. Leyland

**Affiliations:** MRC/CSO Social and Public Health Sciences Unit, University of Glasgow, Glasgow, UK

**Keywords:** Ethnicity, socioeconomic inequalities in health, deprivation, self-rated health, immigrants, minority health

## Abstract

**Objectives:**

We compare rates of ill health and socioeconomic inequalities in health by ethnic groups in Scotland by age. We focus on ethnic differences in socioeconomic inequalities in health. There is little evidence of how socioeconomic inequalities in health vary by ethnicity, especially in Scotland, where health inequalities are high compared to other European countries.

**Design:**

A cross-sectional study using the 2011 Scottish Census (population 5.3 million) was conducted. Directly standardized rates were calculated for two self-rated health outcomes (poor general health and limiting long-term illness) separately by ethnicity, age and small-area deprivation. Slope and relative indices of inequality were calculated to measure socioeconomic inequalities in health.

**Results:**

The results show that the White Scottish population tend to have worse health and higher socioeconomic inequalities in health than many other ethnic groups, while White Polish and Chinese people tend to have better health and low socioeconomic inequalities in health. These results are more salient for ages 30–44. The Pakistani population has high rates of poor health similar to the White Scottish for ages 15–44, but at ages 45 and above Pakistani people have the highest rates of poor self-rated health. Compared to other ethnicities, Pakistani people are also more likely to experience poor health in the least deprived areas, particularly at ages 45 and above.

**Conclusions:**

There are statistically significant and substantial differences in poor self-rated health and in socioeconomic inequalities in health between ethnicities. Rates of ill health vary between ethnic groups at any age. The better health of the younger minority population should not be taken as evidence of better health outcomes in later life. Since socioeconomic gradients in health vary by ethnicity, policy interventions for health improvement in Scotland that focus only on deprived areas may inadvertently exclude minority populations.

## 1 Introduction

There is growing international interest from both academics and policy makers in the relationship between health and ethnicity (Kim et al. 2013; Disney et al. 2017). This partly reflects increasing trends in migration; according to the United Nations (United Nations 2017) the per cent of migrants from total population in high-income countries rose from 9.6% in 2000 to 14% in 2017. In addition, there is a growing understanding that for sustainable development inequalities have to be reduced, and vital information should be collected and analysed by different population groups, such as gender, ethnicity, age, disability, income and so forth (United Nations 2015).

Scotland and the UK are no exception to these trends and considerable strides have been made in understanding health outcomes for minority groups. The Scottish Health and Ethnicity Linkage Study (SHELS) linked the 2001 census to health data (Bhopal et al. 2011) and has since looked at a number of health outcomes, including mortality (Katikireddi et al. 2018), cancer (Bhopal et al. 2012d), stroke (Bhopal et al. 2012b), respiratory diseases (Bhopal et al. 2015), chest pain and angina (Bhopal et al. 2012c). In addition, the Information Services Division (ISD), the analytical arm of the NHS National Services Scotland, and the Scottish Government have published analysis of health outcomes and use of health services by ethnicity (The Scottish Government 2015; ISD Scotland 2017b). Efforts have also been made to improve data collection, such as increasing completeness of coding ethnicity on hospitalization records (ISD Scotland 2017a) and including ethnicity on mortality records in Scotland (Christie 2012).

While in most countries minority populations tends to be in poorer health, in Scotland research suggests that the White Scottish population suffers from lower life expectancy (Gruer et al. 2016) and higher mortality (Bhopal et al. 2018) than other ethnicities. The results are more complex with respect to specific diseases, and other research has highlighted high rates of ill health among White Irish (Bhala et al. 2016) and South Asian populations (Sheikh et al. 2016). There is considerable debate as to what causes these differences, including the unequal distribution of multiple resources such as wealth and power (‘fundamental causes’ approach) (Scott et al. 2013), poor diet and unhealthy habits (smoking, drinking, lack of exercise) (Gruer, Hart, and Watt. 2017), and cultural factors (e.g. misconceptions about treatments and different service utilization patterns) (Lakhanpaul et al. 2014).

We contribute to the study of ethnic differences in health in Scotland by analysing the relationship between ethnicity, self-rated health (SRH) and area deprivation using the most recent 2011 Scottish Census, thereby including a much larger minority population than the 2001 census used previously. We focus on comparing socioeconomic inequalities in SRH by ethnic groups and age using a small-area deprivation measure. Across Scotland, socioeconomic inequalities in health, measured by area deprivation, have increased (Brown et al. 2019) and are high compared to the rest of Europe (Seaman, Leyland, and Popham. 2016). However, based on previous evidence (Becares et al. 2012), we expect different socio-economic gradients in health between ethnic groups. This may occur if factors contributing to ill health, such as those related to the lived environment, are different or have a different impact on health between ethnicities. If this is the case, then policies aimed at improving health and reducing socioeconomic inequalities in health based on the majority population are less likely to be effective in improving the health of minority populations.

### 1.1 Health and ethnicity in scotland and elsewhere

The research on health and ethnicity in Scotland has broadly come to either of two main conclusions: (1) the majority White Scottish population suffers poorer health than most other ethnicities, or (2) the South Asian population has a higher risk of ill health and mortality from specific diseases. Recent work has found that White Scottish men and women experience higher rates of cancer (Bhopal et al. 2012d), higher mortality (Bhopal et al. 2018), including preventable and amenable mortality (Katikireddi et al. 2018), lower life expectancy (Gruer et al. 2016), and worse self-rated health (The Scottish Government 2015) compared to most other larger ethnic groups. For example, both men and women of Asian and other White origin (except White Irish) can expect to live 2–6 years longer than White Scottish (Gruer et al. 2016). The rates for all first cancer diagnosis are twice as high for White Scottish compared to some minority groups, leading the researchers to conclude that the ‘Scottish effect’ – the unexplained worse health in Scotland compared to other parts of the UK – does not apply across all of Scotland’s ethnic groups (Bhopal et al. 2012d).

However, a few ethnic groups, such as the White Irish, often experience poor health similar to that of the White Scottish (Bhopal et al. 2012d; Gruer et al. 2016). White Irish men and women experience the highest risk for alcoholic liver disease (Bhala et al. 2016) in Scotland. Other research has found that the South Asian population has worse health compared to the White Scottish. For example, Pakistani have high rates of heart failure (Bhopal et al. 2012a) and coronary heart disease (Millard et al. 2012). The risk of avoidable hospital admissions is higher for Pakistani, Indian and Bangladeshi men compared to White Scottish men (Katikireddi et al. 2018). The relative risks for first all-respiratory disease hospitalization or death are higher for Indian, Pakistani and other South Asian men and Pakistani and other South Asian women compared to White Scottish (Bhopal et al. 2015). There is also evidence that compared to White Scottish, Pakistani and other South Asians suffer from a higher risk of some gastrointestinal hospitalizations and deaths (Cezard et al. 2016). Finally, all South Asian groups also had higher rates of first asthma hospital admission and death compared to White Scottish (Sheikh et al. 2016). These findings persist when accounting for socio-economic position (e.g. education and area deprivation). Together these works highlight that, in Scotland, ethnic differences in ill health are disease dependent. For this reason, the factors underlying ill health are also likely to be different and are a subject of continuing debate (Gruer et al. 2016).

We also know that ethnic differences in health are already present in infants and children (Parslow et al. 2009; Knowles et al. 2016). The results typically suggest that minority children have poorer health, e.g. Black and Asian infants experience higher rates of congenital heart defect compared to the White population in England and Wales (Knowles et al. 2016). However, it is not well known whether these differences in health remain constant, widen or narrow over the life course. In the USA minorities often have worse health outcomes at early life, and for some groups (Blacks) the health disadvantages increase with age, while for others (Hispanics) the health outcomes converge with the Whites (Haas and Rohlfsen 2010). But in the UK and Scotland there is little evidence on the health trajectories of different ethnicities.

### 1.2 Socioeconomic inequalities in health

With respect to socioeconomic inequalities in health, the evidence suggests a differential relationship across ethnic groups (Chandola 2001; Becares et al. 2012). In England, area deprivation had a greater and a more detrimental effect on the health of White British people compared to some minority groups – while an increase in area deprivation translated into an increase in the probability of poor self-rated health for Bangladeshi, Indian and Pakistani people, the slope was shallower than that of the White British group (Becares et al. 2012). For Black African and Black Caribbean groups there was almost no relationship between area deprivation and health. Research in Scotland has also found that area deprivation and socioeconomic status (SES) have a different impact on health across ethnic groups (Fischbacher et al. 2014). The comparison of eight individual, household and area level measures of deprivation across 10 ethnic groups showed that the impact of SES on cardiovascular disease was generally greater among Whites compared to South Asians, with education having some of the more consistent effects on health (Fischbacher et al. 2014).

It has also been previously recorded that socioeconomic inequalities in health tend to be highest among the working age population (ages 30–60) (Norman and Boyle 2014), but again, we do not know whether this applies across ethnicities. The generally better health of young working age (economic) migrants could lead to reduced socioeconomic inequalities in health among the working age population for some ethnicities, as has been suggested by research in Canada (Khan et al. 2017).

Previous research has found ethnic differences in health, and in socioeconomic inequalities in health, in Scotland. The research examining socioeconomic inequalities in health by ethnicity and age is, however, more limited. This paper analyses the most recent population data available in Scotland, including a larger minority population than previous work. The aim is to quantify self-rated ill health in ethnic groups by age and to compare socioeconomic inequalities in health by ethnicity and age, a subject with substantial policy implications, but which few researchers have thus far explored.

## 2 Materials and methods

### 2.1 Study population

Specially commissioned aggregate data tables from the 2011 Scottish Census were obtained from the National Records of Scotland (National Records of Scotland 2019) and released at the 2001 datazone level (population mean = 815, sd = 275). Aggregate data tables from the census are relatively easily available and do not require comprehensive data access applications or secure infrastructure, such as safe havens. Tables undergo statistical disclosure control (SDC) and are then made accessible to the public online. This allows us to provide baseline results from the most recent census with little cost and significantly faster than applying for individual level data. Our study population was split into 5-year age groups up to 85 and older (for reasons of SDC, ages 0–9 were combined) and includes 13 ethnic groups (ordered from largest to smallest): White Scottish, White British, other White, White Polish, White Irish, Pakistani, Chinese, Indian, African, other Asian, mixed, other, and Caribbean or Black.

Similar tables were also commissioned for the 2001 census, but the much smaller size of minority populations (11.9% non-White Scottish in 2001; 16.0% in 2011) meant that the data were not available for finer grained ethnic groups. For this reason, the analysis focuses on the 2011 data and results for 2001 are available with the data and replication materials from the University of Glasgow data management website <http://dx.doi.org/10.5525/gla.researchdata.629>.

The size and age profile of ethnic groups varies considerably in Scotland and has also changed dramatically in recent decades. Between the 2001 and 2011 censuses the Scottish population increased by over 200,000 people to 5.3 million and is now more diverse than it was in 2001. (See Table S.1 for population distribution.) In 2001 just over 78,000 people identified as other White, but in 2011 this was more than double at almost 168,000 (including 61,000 people who identified as White Polish). There have been similar increases among all non-British ethnic groups. Minority populations are also much younger – while about 60% of White Polish are aged 20–39 and only 2% are over 65, among White Scottish these percentages are 24% and 18%.

Due to SDC the data did not include a breakdown between genders, but previous research has found that ethnic differences in health generally apply in a similar manner for both men and women (Bhopal et al. 2012b; Millard et al. 2012; Bhopal et al. 2015; Gruer et al. 2016). For most ethnic groups, this has also been shown to be the case for poor general health and limiting long-term illness, but the effects of ethnicity on SRH are not always of the same magnitude or statistically significant for both genders (Mindell et al. 2014; Becares 2015).

### 2.2 Health outcomes

We used two measures of SRH: poor general health and limiting long-term illness (LLTI). The poor general health measure combines all those who rated their health bad or very bad. The long-term illness measure includes those who said that their day-to-day activities were limited a little or a lot due to a long-term condition. (See the census metadata website <http://www.scotlandscensus.gov.uk/variables-classification> for exact questions.)

We do not use an objective measure of health, but in England, poor self-rated health has been found to associate with objective measures of health across ethnic groups (Chandola and Jenkinson 2000) and the use of this indicator in health and ethnicity research is common (Smith and Grundy 2011; Mindell et al. 2014). The two separate questions used here should also contribute to the robustness of the results.

Ill health is presented as standardized rates per 1000 population, using the 2013 European Standard Population (Eurostat 2013) and are analysed by age and small-area deprivation.

### 2.3 Small-area deprivation measures

Area deprivation is measured at datazone level using a census-based measure including indicators of unemployment, tenure, educational qualifications and National Statistics Socioeconomic Classification (NS-SEC) (Allik et al. 2016). Datazones are divided into population-weighted quintiles ranked from 1 (least deprived) to 5 (most deprived). It has been argued that area deprivation measures may be driven by the majority White population and are unable to account for individual deprivation for minority groups (Smith 2000). A study in England (Baker, Mitchell, and Pell 2013) did find lower agreement between the English Index of Multiple Deprivation (IMD) and individual measures of SES among some minority groups, but the ability of IMD to positively predict individual deprivation among minority groups was not worse compared to the White population.

We are also not relying on a single indicator of deprivation. The small-area measure used here is a combination of four different variables and that should increase its reliability for different ethnic groups. In addition, we were able to repeat the analysis with the same results using deprivation quintiles calculated from the income domain of the 2012 Scottish Index of Multiple Deprivation (SIMD) (The Scottish Government 2012) and the 2011 Carstairs score (Brown et al. 2014). There are also benefits to using area level measures of deprivation compared to individual level SES. Area-based measures are better able to include the whole population (regardless of age and gender), while individual level employment or education indicators of SES may sometimes only be available for the majority male working age population (Tobias and Cheung 2003).

### 2.4 Statistical methods

Since the proportion of older people is fairly small for many minority groups the results generally focus on ages 0 to 64. Socioeconomic inequalities in health are measured using the slope index of inequality (SII) and the relative index of inequality (RII), the latter calculated as SII divided by the mean level of population health (Regidor 2004). The confidence intervals (CIs) for both measures are calculated using a multinomial simulation method (Lumme et al. 2015). The results are statistically significantly different when the confidence intervals for the standardized rates, SII and RII do not overlap. More detailed age specific analysis of inequalities was only possible for the 10 biggest ethnic groups as there are insufficient data to provide detailed breakdown of self-rated health by ethnicity, age and deprivation for the smaller ethnic groups. The statistical analysis was conducted using R version 3.50 (R Core Team 2018) and the R package SocEpi for health inequalities research.

## 3 Results

### 3.1 Self-rated health by ethnicity and age

Table 1 shows the direct age standardized rates of ill health together with the 95% CI for ages 0–64 by the 13 ethnic groups ordered by population size, from highest to lowest. White Scottish, Pakistani and those of mixed and other ethnicity are most likely to rate their health poor and report a long-term condition that limits their day-to-day activities, while those of White Polish, Chinese and African background are least likely to report health problems. The White British, White Irish, Indian, other White and other Asian also report significantly fewer health problems than the White Scottish and Pakistani.

Differences in poor health between ethnicities are quite large. Only 77 (95% CI = 74–80) White Polish and 72 (CI = 68–76) Chinese in every 1000 report having a long-term health condition while for Scottish and Pakistani ethnicities the rates are 135 (CI = 134–135) and 181 (CI = 176–186) per 1000 respectively. Overall, the ethnic groups that are more likely to report poor general health are also more likely to report limiting long-term conditions. The two slight exceptions are White British and those in the residual other category. Compared to White Polish, the White British are more likely to report long-term conditions, but report similarly low levels of poor general health.

The results in Table 1 do not, however, hold for all age groups. Some ethnicities are more likely to rate their health poor at younger ages and others at older ages (see Table S.3). For example, at ages 15–29 and 30–44 people of Indian background report low levels of poor SRH similar to White Polish and Chinese, but at ages 45–59 they report long-term conditions and poor general health more often than many other ethnicities. For White British this age-SRH relationship is almost opposite – compared to other ethnicities they are more likely to report poor health and long-term conditions up to ages 30–44, but for ages 45 and up, the White British are among the least likely to report poor general health and long-term conditions. White Polish, Chinese and Africans report limiting long-term conditions and poor general health less often compared to most ethnicities across the working ages between 15 and 59. White Scottish, those of mixed background and particularly Pakistani are more likely than other ethnicities to report poor SRH across the different age groups. Notably, for Pakistani, the rates of ill health are similar to the White Scottish up to ages 30–44, but are significantly higher than among any other ethnic group for ages 45–59 and 60–74.

For ages 75 and above the rates of ill health become more similar across ethnic groups. Minority groups that had very low levels of poor SRH at younger ages have similar (e.g. African, Indian) or sometimes worse (e.g. White Polish) levels of ill health at ages 75 and over compared to the majority White Scottish. Despite the increasingly similar rates of ill health at older ages across all ethnicities, Pakistani population still reports the worst SRH for those aged 75 and over.

These data show that many minority groups (e.g. White Polish, Chinese, African) experience much better self-rated health in most age groups than the White Scottish majority, who have some of the worst self-rated health in Scotland. However, these health benefits among the minorities are mostly evident among the working aged adults and the differences dissipate for those aged 60 and above. The health of the White Scottish is also worse than that of the White British and Irish living in Scotland. Other minority groups, like those of mixed and particularly of Pakistani origin, have poor self-rated health similar to the White Scottish. Notably, the Pakistani population reports by far the worst SRH across all ethnic groups from the middle age (45 and above).

### 3.2 Socioeconomic inequalities in health by ethnicity and age

**Figure 1:**
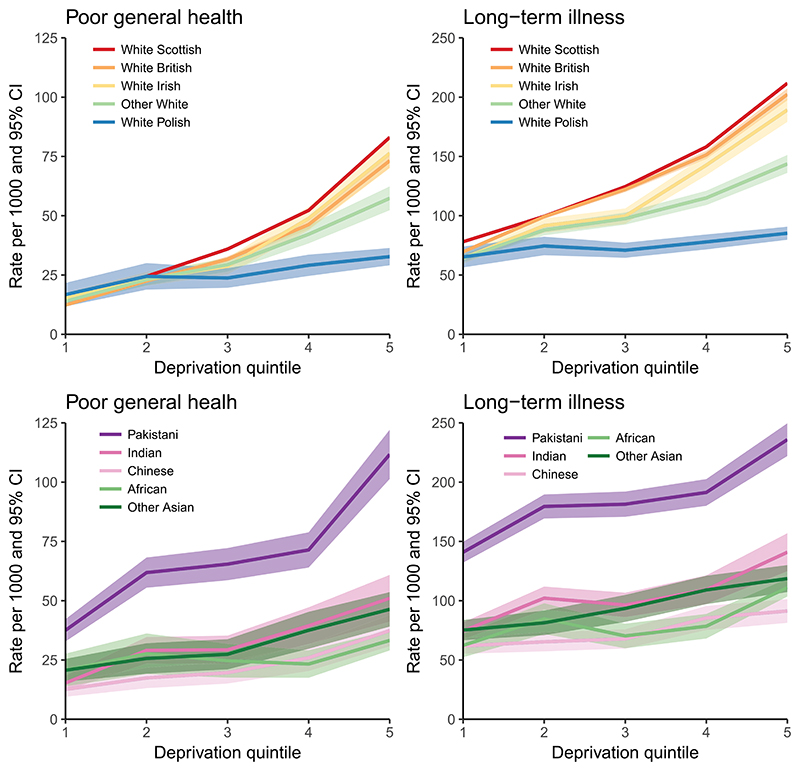
Self-rated health by deprivation quintiles (1 – least, 5 – most deprived) for 10 larger ethnic groups for ages 0–64, 2011.

Some of the variation in the levels of self-rated health between ethnic groups may stem from differences in area deprivation. In 2011 the White British, Irish, other White, Indian and people of a mixed background were more likely to live in the least deprived areas in Scotland, while other ethnicities, particularly White Polish, African and Caribbean or Black, were disproportionately more likely to live in deprived areas (see Table S.2 for details). When we look at poor self-rated health by deprivation some of the differences in health outcomes between ethnicities disappear. Figure 1 shows the rates of poor general health and LLTI for the 10 largest ethnic groups by deprivation quintiles. While across Scotland White British and White Irish reported better SRH compared to White Scottish, the three ethnic groups have similar levels of ill health across the five deprivation quintiles.

The figure also shows that deprivation appears to have a much stronger and detrimental effect on the health of the White British, Irish and Scottish, compared to other Whites and particularly White Polish. For the White Polish group the rate of poor general health is 17 (95% CI = 12–22) in the first and 33 (CI = 29–36) per 1000 in the most deprived fifth quintile. For the White British, Irish and Scottish the rates are roughly 12–16 in the first, but 73–83 per 1000 in the fifth quintile (for Scottish 15.5 CI = 15.2–15.8 in the first and 83.0 CI = 82.4–83.7 in the fifth quintile). The rate of LLTI for the White Polish is statistically significantly lower compared to the White Scottish, British and Irish in all quintiles, except in the least deprived quintile. For general health these differences are significant for the more deprived fourth and fifth quintiles. White Polish do not appear to experience the same detrimental effects of deprivation on health as do the White Scottish, British and Irish.

The socioeconomic gradients in SRH are also shallow for people of Indian, Chinese, African and other Asian origin in comparison to the majority White Scottish. For Pakistani, however, the gradient is steep. Notably, the rates of both poor general health and LLTI are significantly higher among Pakistani across all deprivation quintiles compared to the other nine bigger ethnic groups. Among Pakistani, the rate of LLTI in the least deprived first quintile is 141 (95% CI = 132–150), which is comparable to the rate of LLTI among the Scottish (158, CI = 157–159) in the deprived fourth quintile, and to other Whites (144, CI = 136–151) and Indians (141, CI = 125–157) in the most deprived fifth quintile. The rates of LLTI among White Polish and Chinese in the most deprived areas are significantly lower than for Pakistani in the least deprived areas.

Table 1 quantifies what was evident in Figure 1 by showing the SII and the 95% confidence intervals for both measures of health for all 13 ethnic groups for ages 0–64. The SII can be interpreted as the estimated absolute difference in the level of poor self-rated health between people in the least and most deprived areas. The White Scottish group have the highest absolute socioeconomic inequalities in health, followed by White British and Irish. Pakistani have high absolute socioeconomic inequalities in general health (similar to the White Scottish), but the SII for LLTI is lower among Pakistani than for the White Scottish, British and Irish. White Polish have the lowest absolute inequalities in health, followed by people of African, Chinese, Indian and other Asian descent. The differences in the SII between the ethnic groups with the lowest (e.g. White Polish, Chinese, African) and those with the highest inequalities (e.g. White Scottish, British, Irish and Pakistani) are statistically significant. People of Caribbean or Black descent have low socioeconomic inequalities in health while mixed and other ethnicities have average to high socioeconomic inequalities in health, but the CIs for these ethnic groups are wide.

**Figure 2:**
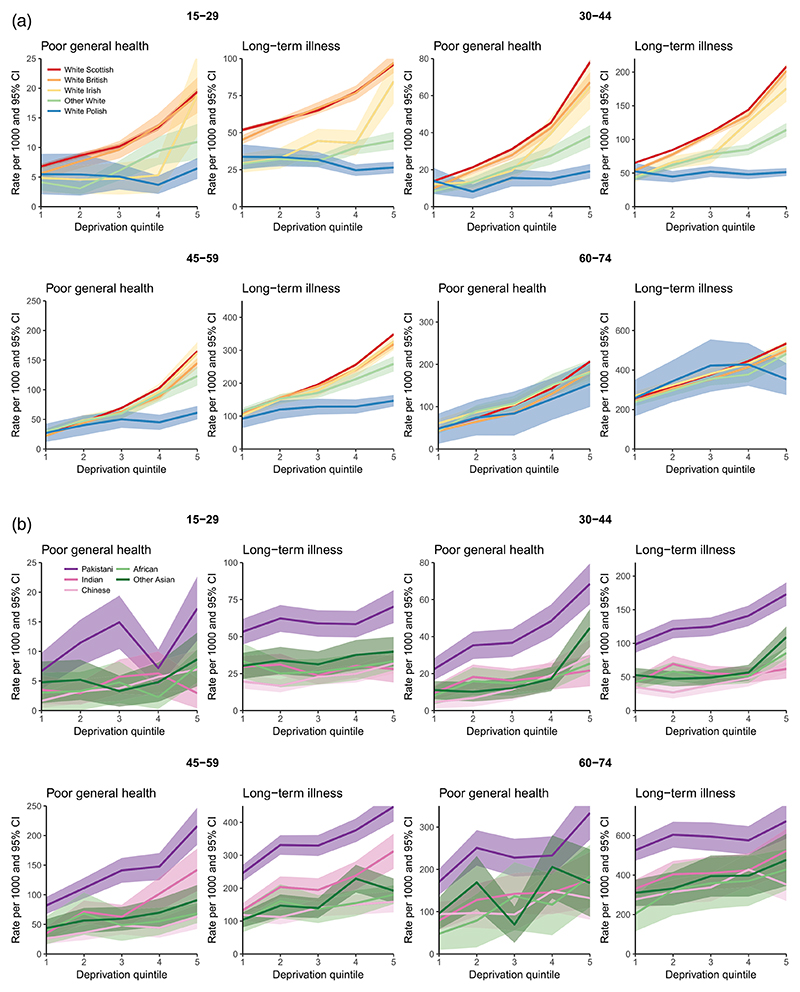
(a) Self-rated health by deprivation quintiles (1 – least, 5 – most deprived) and age for five White ethnic groups, 2011. (b) Self-rated health by deprivation quintiles (1 – least, 5 – most deprived) and age for Asian and African ethnic groups, 2011.

For the 10 biggest ethnic groups we were also able to look at socioeconomic inequalities in health by 15-year age groups. This shows that the relationship between deprivation, health and ethnicity varies considerably by age group. Figure 2(a,b) replicate the previous plot for ages 15–29, 30–44, 45–59 and 60–74 separately. For the White Scottish, British and Irish groups the gradients are fairly similar across ages, but for the other ethnicities these change quite markedly. Between the ages 15–29, 30–44 and 45–59 White Polish, African and Chinese people have very shallow socioeconomic gradients in health, and only at ages 60–74 do these gradients becomes more notable. People of other White and Indian origin also have shallow socioeconomic gradients among the younger working ages (15–29 and 30–44), but for these two ethnic groups the gradient becomes steeper and similar to the White Scottish, British and Irish at ages 45 and above.

For Pakistani, the socioeconomic gradients in SRH are not steeper than for White Scottish, British and Irish. However, Pakistani have higher rates of poor health than the other ethnicities at the same deprivation quintiles, particularly for ages 45–59 and 60–74. The rate of LLTI for Pakistani aged 45–59 in the least deprived first quintile is 246 (CI = 221–271), which is similar to the rate of LLTI among those of Indian (239, CI = 195–282), White Scottish (256, 254=258), White British (241, CI = 232–249) and White Irish (242, CI = 219–264) background in the deprived fourth quintile.

**Figure 3:**
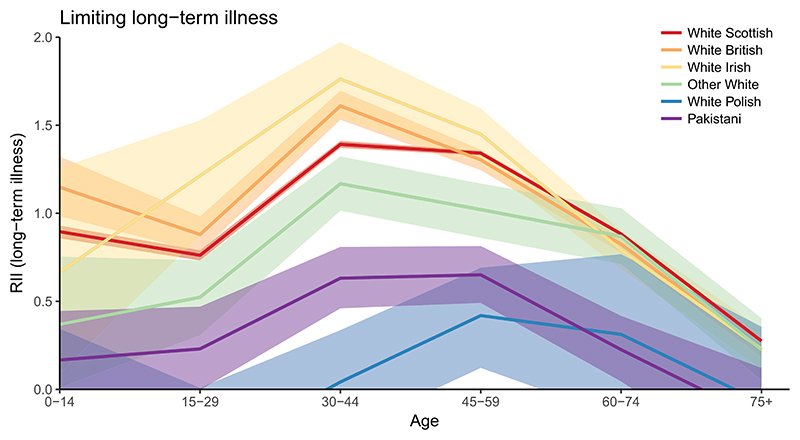
Relative index of inequality for LLTI by age (six larger ethnic groups), 2011.

As an absolute measure, the SII is sensitive to the mean level of population health, making comparisons of inequalities across age groups difficult. To quantify socioeconomic inequalities in health by age groups, Figure 3 shows the RII with 95% CIs for the six biggest ethnic groups. Results for the Indian, Chinese, other Asian and African groups were excluded from the plot as the CIs were too wide. A RII value of zero indicates no inequalities. A value of one suggests that the level of poor self-rated health between the people in the least and most deprived areas is equal to the mean level of poor health. That is, the level of poor health is about 50% above average in the most deprived areas and 50% below average in the least deprived areas. The upper bound of RII is two, but in certain circumstances this can be exceeded.

For most ages the White Scottish, British and Irish have significantly higher relative socioeconomic inequalities in health compared to the other Whites, Pakistani and especially White Polish. The differences are greatest at ages 30 to 44 – the socioeconomic inequalities among Pakistani and White Polish for LLTI are 0.63 (CI = 0.46–0.81) and 0.04 (CI = -0.25-0.34, i.e. not significant) respectively, but for White Scottish the RII is 1.40 (CI = 1.38–1.41), for White British 1.61 (CI = 1.53–1.70), and for White Irish 1.76 (CI = 1.54–1.97).

Socioeconomic inequalities in health tend to be highest for working age populations (aged 30–59) and this is most evident for the White Scottish, British and Irish. For Pakistani the differences in relative inequalities in health across age groups are less notable and generally fairly low, but it should be kept in mind that the level of ill health among Pakistani is higher compared to the other ethnic groups, reducing relative inequalities. For White Polish the relative inequalities in LLTI are only statistically significant at ages 45–59. The patterns seen in Figure 3 are similar, though less pronounced, for poor general health (see Figure S.1).

## 4 Discussion

The analysis of the 2011 census data shows that the relationship between ethnicity, selfrated health and socioeconomic inequalities in health is not straight forward. As previous research (Bhopal et al. 2012d; Gruer et al. 2016; Bhopal et al. 2018; Katikireddi et al. 2018), we find that White Scottish experience poorer health compared to many minority populations, including White British, White Irish, White Polish and other White groups. Similarly to other previous work (Bhopal et al. 2012a; Millard et al. 2012; Bhopal et al. 2015; Cezard et al. 2016; Katikireddi et al. 2018), we also find that the Pakistani population also has high rates of poor ill health, especially for ages 45 and above. Overall, while we have used self-rated health measures, our results are similar to the cited studies that have used objective measures of health, such as hospitalizations and mortality.

Unlike most previous work, we were also able to compare health outcomes for the different ethnicities across age and area deprivation, revealing further complexities. For example, we find that some minority groups (e.g. Indian) are more likely than other ethnicities to experience poor self-rated health after the age of 45, while having relatively low levels of poor health prior to this. White British on the other hand experience some of the best self-rated health from the age of 45, while being more likely to rate their health poor prior to that.

Some of the ethnic differences in health are reduced or disappear when small-area deprivation is accounted for, and this is consistent with previous research (Mindell et al. 2014). For example, White British and Irish have similar self-rated health to White Scottish at any given deprivation quintile. But other differences remain, most notably for White Polish and Pakistani. Socioeconomic inequalities in health are among the lowest for White Polish, who have low rates of poor SRH even in the most deprived areas. This is particularly true for the young and working age people aged 15–59. Pakistani, on the other hand, have higher levels of poor health compared to other groups, including in the least deprived areas, and this is more the case for those aged 45 and above. Our results for people of Pakistani and also of Indian origin echo research that has found an earlier onset of cardiovascular disease among South Asians (George et al. 2017), worse self-rated health (Evandrou et al. 2016) and higher per cent of long-term illness (Smith and Grundy 2011) among older and middle-aged people of Indian, Pakistani and Bangladeshi origin.

There can be many explanations as to why the association between area deprivation and health differs between ethnicities. Indicators of area deprivation may not accurately capture the life circumstances of some ethnicities and reflect cultural norms rather than deprivation or health. This means that what is observed in Figures 1–3 is not so much difference in socioeconomic inequalities in health between ethnicities, but rather an inability to understand and measure deprivation (Braveman et al. 2005). However, others have argued area level measures can perform relatively well in picking up deprivation for different ethnic groups in England (Baker, Mitchell, and Pell 2013) and in Scotland the SIMD had a similar effect on cardiovascular disease across ethnic groups compared to individual educational level and highest household qualification (Fischbacher et al. 2014).

We also tested the robustness of the results by using two additional measures of deprivation (Carstairs score and SIMD income domain) and found that the patterns in socioeconomic inequalities in health were confirmed (see Supplement Figure S.2). White Scottish, British, Irish, Pakistani, and those of mixed and other background have the highest, and White Polish, Chinese and African the lowest absolute inequalities in health. The differences in area deprivation measures cannot explain away ethnic differences in socioeconomic inequalities in health.

It is also possible that behaviours that have an impact on health (e.g. exercise, diet, smoking, drinking) vary across ethnicities, and do so even after controlling for deprivation. Low levels of physical activity among South Asians (Fischbacher, Hunt, and Alexander 2004), higher levels of obesogenic lifestyle among children of Black and Asian origin (Falconer et al. 2014) and higher rates of obesity among Black African and Caribbean children (Karlsen et al. 2014) have been reported, while smoking appears more common among White British compared to some minority groups (Karlsen, Millward, and Sandford 2012). These works indicate that among White British health behaviours are more closely linked to deprivation, leading to higher socioeconomic inequalities in health. Among some minorities health behaviours have a weaker relationship with deprivation contributing to lower socioeconomic inequalities in health. This would mean that socioeconomic inequalities in health are truly different across ethnic groups.

The healthy migrant effect can also explain why some minority groups have lower levels of poor health and low socioeconomic inequalities in health, particularly for younger working age adults (Elstad 2016; Khan et al. 2017). We know most of the young minority population (aged 15–44) in Scotland to be recent migrants and thus the results for working age White Polish and Chinese are in line with this theory. However, researchers have found that immigrants’ health, health behaviours and use of health services often converge with the native population over time (Leão et al. 2009). Convergence may mean both improved and worsened health outcomes for migrants – in Norway hospitalization rates increased with duration of stay for economic migrants, but decreased for refugees (Elstad 2016). Previous UK research also suggests that the health and health behaviours of migrants may converge with the White British (Wang and Mak 2018) and health outcomes may be worse for migrants who have spent more time in the UK (Kearns et al. 2016). We cannot say if the health of White Polish or Chinese will deteriorate over time to converge with the White Scottish, but our work does show that White Polish do not have a health advantage over White Scottish at ages 60 and above.

To summarize, while we find that some minority ethnic groups have better SRH and lower socioeconomic inequalities in health compared to the White Scottish majority, this often applies only to younger age groups and at older ages this can be the reverse. Our work cannot say if these results are driven by acculturation, generational differences or length of residence in the UK. For this reason, we see our work as evidence that more detailed research on this topic should be undertaken, such as studying the health trajectories of minorities over time while including evidence of migration histories. Since the reasons, time and age at migration vary both between and within ethnic groups, a large population-based study is required to understand the various paths that lead to the different levels of ill health and socioeconomic inequalities in health for the different ethnic and age groups. This could be achieved by linking the 2011 Scottish census with mortality and/or morbidity records, together providing details on ethnicity, country of birth, length of residence in the UK and objective health outcomes.

### 4.1 Strengths and limitations

Some limitations of this study affect much of the research in this field, these include restricting comparisons to a few large ethnic groups, large standard errors and a limited number of covariates. For the smaller ethnic groups we were not able to provide robust results by age groups. For disclosure reasons we have also been unable to distinguish between ethnicity, country of birth and length of residence in the UK. This does not allow us to investigate whether differences in health between ethnicities eventually converge with time lived in Scotland or the UK. Similarly, we could not include other individual level covariates, such as individual socioeconomic position, family status or any information about exercise or diet. Again, this is not very unusual in this field of research. The small number of minorities in most samples, and even the census, is often not sufficient to include a rich set of covariates or provide a detailed estimation of cause specific mortality or health conditions. The findings tend to generally be more robust for broader disease categories (all-cause mortality, all cancers or combining hospitalizations and deaths for the same cause) and for bigger ethnic groups (Bhopal et al. 2012b; Bhala et al. 2016).

We have used aggregate data and a census-based small-area deprivation measure to capture socioeconomic inequalities in health. This means that our work carries the risk of ecological fallacy – if small-area deprivation is an inaccurate indicator of individual level SES for minority ethnic groups, then the socioeconomic gradients of ill health may be misestimated.

The strengths of this work include its population wide coverage, the use of several measures of area deprivation and the use of the most recent 2011 census data. The latter is quite important as previous work, such as research from the SHELS (Bhopal et al. 2011), is based on the 2001 census. Since the minority population in the 2011 census is much larger, we were able to provide evidence for the 10 largest ethnic groups by age, something that has not received much attention before. As the data come from a single source, it also does not have the risk of wrongly estimating the numerator or the denominator in minority groups (ISD Scotland 2017b). The paper addresses an increasingly important issue and shows considerable complexity in the relationship between health, socioeconomic deprivation and ethnicity.

## 5 Conclusions

Our study has examined ethnic differences in ill health and in socioeconomic inequalities in health, an increasingly important public health focus. We have made two important contributions that previous work has not looked at in great detail. First, the rate of ill health at any age group varies considerably across ethnic groups. There may be a higher risk of poor health for the Pakistani and Indian population from around the age of 45. Also, while immigrant (or minority) youth are in better health, their health trajectories in later life are not well known and may converge with the White Scottish population with years of residence. This means that the relatively better health of minority youth should not be taken as evidence of good health in later life. Secondly, socioeconomic gradients in ill health are different for some minority groups compared to the White Scottish, British and Irish populations. Policy interventions for improving health that focus only on deprived areas may inadvertently exclude minority populations, such as Pakistani, who are more likely than the White Scottish, British and Irish to experience ill health in the least deprived areas.

## Supplementary Material

Supplementary Material

## Figures and Tables

**Table 1:** Self-reported health and slope index of inequality by ethnicity for ages 0–64, 2011.

Ethnicity	Poor general health					Long-term illness			
	Rate	95% CI	SII	95% CI		Rate	95% CI	SII	95% CI
White Scottish	42.2	42–42.4	82.3	81.6–83		134.7	134.4–135.1	164.4	163.1–165.7
White British	28.3	27.8–28.8	61.8	59.6–64.1		110.3	109.3–111.4	139.9	135.6–144.5
White Other	28.5	27.2–29.7	50.8	45.4–56.1		93.3	91.2–95.5	90.4	81.5–99.5
White Polish	27.3	25.3–29.3	17.3	7.9–26.7		77.1	74.1–80.1	22.0	6.5–37
White Irish	34.8	33.1–36.5	69.0	63.4–74.9		109.4	106.4–112.5	143.3	131.9–154.5
Pakistani	65.5	62.4–68.5	76.3	62.8–88.8		180.8	176–185.6	98.0	77.1–117.3
Chinese	21.1	19–23.1	29.0	19.7–38		71.9	68.2–75.6	39.3	23.2–56.7
Indian	28.6	26.1–31.2	39.4	28.8–50.8		97.3	92.8–101.9	65.9	44.7–86
African	27.9	25.2–30.6	14.3	1.7–28.1		88.0	83.6–92.5	61.2	38.1–87.6
Other Asian	30.6	27.8–33.5	32.3	19.4–44		93.8	89.1–98.5	58.3	37.8–78.4
Mixed	44.9	40.2–49.6	67.3	48.8–90.4		143.0	135.8–150.1	113.8	78.4–147.1
Other	42.4	38.1–46.7	75.4	59.1–91.2		118.3	111.4–125.2	114.7	87.8–143.5
Caribbean or Black	37.9	32.3–43.5	45.5	19.1–70.9		118.7	109.4–127.9	76.0	40.7–114.7
