# Supplementary material for: Differences in ill health and in socioeconomic inequalities in health by ethnic groups: a cross-sectional study using 2011 Scottish census
Source: Ethn Health. Author manuscript; Available in PMC 2023 Feb 27. (PMC7614248; doi:10.1080/13557858.2019.1643009)
Supplement: Supplementary Material [file EMS158439-supplement-Supplementary_Material.pdf]

Check for updates

Received: 12 May 2020 | Revised: 31 July 2021 | Accepted: 22 August 2021

DOI: 10.1002/pst.2168

MAIN PAPER

WILEY

A two-stage drop-the-losers design for time-to-event outcome using a historical control arm

Rachid Abbas[1,2] | James Wason[3,4] | Stefan Michiels[1,2] | Gwénaël Le Teuff[1,2]

[1]Biostatistics and Epidemiology Department, Gustave Roussy, Villejuif, France

[2]Oncostat U1018, Inserm, Université Paris-Saclay, Ligue contre le Cancer, Villejuif, France

[3]Population Health Sciences Institute, Newcastle University, Newcastle Upon Tyne, UK

[4]MRC Biostatistics Unit, University of Cambridge, Cambridge, UK

Correspondence
Rachid Abbas, Biostatistics and Epidemiology Department, Gustave Roussy, Villejuif, France.
Email: abbas.rachid@gmail.com

Abstract

Phase II immuno-oncology clinical trials screen for efficacy an increasing number of treatments. In rare cancers, using historical control data is a pragmatic approach for speeding up clinical trials. The drop-the-losers design allows dropping off ineffective arms at interim analyses. We extended the original drop-the-losers design for a time-to-event outcome using a historical control through the one-sample log-rank statistic. Simulated trials featured three arms at the first stage, one at the second stage, nine scenarios, eight sample sizes with 5%- and 10%- nominal family-wise error rate (FWER). A numerical algorithm is provided to solve power calculations at the design stage. Our design was compared with a group of three independent single-arm trials (fixed design) with and without correction for multiplicity. Our design allowed strict control of the FWER at nominal levels while the misspecification of survival distribution and fixed design inflated the FWER up to three times the nominal level. The empirical power of our design increased with the sample size, the treatment effect and the number of effective treatments and dropped when more patients were recruited at the second stage. The fixed design with correction showed comparable power, while our design advantageously included more patients to the most promising arm. Recommendations for future applications are given. By taking advantage of the use of historical control data and a time-to-event outcome, the drop-the-losers design is a promising tool to meet the challenge of improving phase II clinical trials in immuno-oncology.

KEYWORDS

adaptive design, historical control, oncology phase II trials, one-sample log-rank, time-to-event

1 | INTRODUCTION

The number of experimental treatments available in oncology and potentially evaluable in phase II clinical trials has increased with the recent availability of immune- or targeted therapies.[1] For example, preclinical investigations showed the potential of novel treatment strategies targeting specific immune signaling pathways.[2,3] The programmed cell death 1 (PD1) and programmed cell death ligand 1 (PD-L1) blockade was identified as a putative evasion mechanism in advanced solid tumors. Between 2014 and 2018, five immune checkpoint inhibitors targeting the PD-1/PDL1 axis were

© 2021 John Wiley & Sons Ltd.

15391612, 2022, 1, Downloaded from https://onlinelibrary.wiley.com/doi/10.1002/pst.2168 by ${individualUser.givenNames} ${individualUser.surname} - University of Cambridge , Wiley Online Library on [15/11/2022]. See the Terms and Conditions (https://onlinelibrary.wiley.com/terms-and-conditions) on Wiley Online Library for rules of use; OA articles are governed by the applicable Creative Commons License

developed and approved by the Food and Drug Administration for seven tumor types.[4] Future phase II clinical trials will have to evaluate simultaneously more and more experimental treatments to select the most promising treatments that deserve further assessment in confirmatory phase III clinical trials. In the meantime, phase II clinical trials will have to face this challenge with a limited number of eligible patients. This is particularly true for the development of immunotherapy clinical trial programs in pediatric solid tumors, which are rare diseases. For instance, several targetable pathways were identified in osteosarcoma, the most common primary bone tumor, which has less than five new cases per million per year.[5]

Multi-arm multi-stage (MAMS) trials[6] are efficient in evaluating several treatments simultaneously. MAMS trials are a special class of 'adaptive designs' featuring planned opportunities for early termination of one or several arms based on the analysis of accumulating data. As discussed by Parmar et al.[7] from experiences in their institution, multi-arm trials recruited faster than comparable two-arm designs. They reported that the evaluation of treatment in a multi-arm trial was half the cost it would have in a two-arm trial. While comparing to a series of two-arm clinical trials, multi-arm trials allow more treatments to be tested and produce contemporaneous results for all experimental treatments. The MAMS designs involve a multiplicity of null hypotheses and interim analyses that may inflate the overall type I error rate and thus specific analytical methods have been developed to control this error rate.[8–15] The drop-the-losers design,[16] also referred to as 'pick-the-winner', is a special class of MAMS design that allows dropping less promising arms at interim analyses with full control of the overall type I error rate. In practice, the formulation of this design is generally a two-stage design. Adding a supplementary stage has been proven to be efficient when there are at least four experimental arms in consideration.[6] This design was implemented for normal[17] and binary[18] outcomes but not extended to time-to-event outcomes. In addition, the drop-the-losers design has been used with a control arm. For patients with a rare life-threatening disease, the use of a control arm may be unethical or unfeasible.[19,20] Several phase II clinical trial designs with an historical control arm for survival outcomes were proposed: the phase II design with Edgeworth expansion of Sun et al.,[21] the two-stage or optimal two-stage design by Kwak et al.,[22] or the group sequential design of Wu et al..[23] The Kwak's optimal two-stage design is based on the one-sample log-rank test (OSLRT)[24,25] with a procedure restricted to the exponential distribution. Wu et al. provided a modified one sample log-rank test (MOSLRT)[23] which allows the use of more flexible survival distributions. They showed that the MOSLRT was more efficient than the OSLRT and protected well the type I error rate (the OSLRT tended to be conservative even with relatively large sample sizes). However, these designs are limited to the evaluation of a single experimental arm versus an historical control. A strategy to improve the efficiency of phase II clinical trials for rare diseases would be to combine the advantage of the two-stage drop-the-losers design with an historical control, picking up only the best experimental treatment at the interim analysis. Given the high attrition rates observed in phase II clinical trials, the likelihood of having more than one efficient and tolerable experimental treatment is low.[26] Besides, having two experimental arms in a second stage will certainly reduce the speed of the trial. Thus, the selection of no more than one experimental arm at the second stage seems an efficient strategy within the framework of phase II oncology trials.

Motivated by a proposal for a phase II randomized clinical trial evaluating three immunotherapies in relapsing osteosarcoma, we developed a two-stage drop-the-losers design for a time-to-event endpoint (progression-free survival, PFS) with a historical control treatment. In section 2, we describe the methods used to extend the drop-the-losers design for a time-to-event outcome and introduce the one-sample log-rank test. The simulation setting is detailed in section 3 and the results are reported in section 4. In section 5, we discuss our findings and give recommendations for the planning of a phase II clinical trial using a two-stage drop-the-losers design with historical control for time-to-event outcome.

2 | DROP-THE-LOSERS DESIGN FOR TIME-TO-EVENT OUTCOME USING HISTORICAL CONTROL ARM

We proposed an extension of the two-stage drop-the-losers design both for time-to-event outcome and using historical control data. First, a description of the drop-the-losers design extended to time-to-event outcomes using a historical control group is given. Second, the one-sample log-rank test used in this design is defined.

15391612, 2022, 1, Downloaded from https://onlinelibrary.wiley.com/doi/10.1002/pst.2168 by ${individualUser.givenNames} ${individualUser.surname} - University of Cambridge , Wiley Online Library on [15/11/2022]. See the Terms and Conditions (https://onlinelibrary.wiley.com/terms-and-conditions) on Wiley Online Library for rules of use; OA articles are governed by the applicable Creative Commons License

2.1 | Description of the design

A drop-the-losers design is a trial with K experimental arms randomized to a control arm and J interim analyses at which a pre-specified number of experimental arms are dropped. At each interim analyses ($j=1,...,J$), experimental arms are compared to the control arm using all data gathered until then. The decision rule for dropping experimental arms is based on the ranking of statistical tests. Experimental arms with the lowest test statistic (least promising efficacy) and thus lower ranking are dropped. The control arm is kept until the final stage. The number of experimental arms, the number and the timing of interim analyses and the number of arms to be dropped at each interim analyses are all pre-specified before starting the trial. In this paper, we will focus on a two-stage ($J=2$) drop-the-losers design starting with $K=3$ experimental arms and keeping one experimental arm at the second stage which is generally noted as (3:1). In the following, we assume that n_1 patients are allocated to each of the three experimental arms in the first stage, after the interim analysis n_2 patients are allocated to the remaining arm. The values of n_1 and n_2 may differ; we will refer to unequal allocation as the ratio $r=\frac{n_1}{n_2}$.

In settings without contemporaneous control arm, our proposal consists in randomizing patients in three experimental arms assuming that appropriate historical data is available for comparison. To compare these three experimental arms to an historical control arm with a time-to-event outcome we use the hazard ratio (HR), δ_k, to measure the relative efficacy of experimental arm $k=(1,...,3)$ to the historical control h, which is defined as follows:

$$S_k(t)=[S_h(t)]^{\delta_k} \tag{1}$$

In the following, we will assume that the proportional hazards assumption holds. We can define three null hypotheses of the form:

$$H_{0k}:\delta_k\geq 1 \tag{2}$$

The global null hypothesis, H_G, is defined as: $H_G:\delta_1=\delta_2=\delta_3=1$.

Let us introduce the random variable, γ, representing the ranking of the three experimental treatments arms over the whole trial. At the end of the (3:1) drop-the-losers trial, each experimental arm can be assigned to a rank, γ_k, which are permutations of the integers from 1 to 3. Let us denote Z_{jk}, the test statistic comparing the experimental arm k to the historical control data from all data gathered up to the stage j. At the interim analysis, the experimental arm with the highest Z_{1k} is rank 1, the two remaining experimental arms are dropped and for $Z_{1k'}>Z_{1k''}$, the rankings are $\gamma_{k'}=2$ and $\gamma_{k''}=3$. At the final analysis, one experimental treatment remains. If its test statistic, Z_{2k}, is above a threshold value, c, the corresponding null hypothesis, H_{0k}, is rejected meaning that this experimental treatment from arm k is recommended. The threshold value c is chosen in order to control the family-wise type I error rate (FWER). Wason et al.[27] demonstrated that controlling the FWER under H_G at level α suffices to control the FWER at level α or lower for any configuration of true and false null hypotheses (this is called 'strong' control of the FWER).

For a (3:1) drop-the-losers, the probability of recommending a treatment k (to reject H_{0k}) is the probability that treatment k reaches the final stage of the trial, $\gamma_k=1$, and that the final test statistic is above c, $Z_{2k}>c$. In the following, we will use a vector, Z, including the test statistics Z_{jk} ($j\in\{1,2\}$) and ($k\in\{1,...,3\}$) for each arm and each stage: $Z^t=(Z_{11},Z_{21},Z_{12},Z_{22},Z_{13},Z_{23})$. The vector Z is multivariate normal with mean μ and variance–covariance matrix, Σ. In our setting, as there is no shared control group, data from distinct treatment arms are independent and the variance matrix uses the correlation between stages, ρ. For instance, the correlation between stage 1 and stage 2 of the treatment arm 1 test statistics is defined as:

$$\rho_1=\frac{\sqrt{E_{11}}}{\sqrt{E_{21}}}$$

where E_{11} and E_{21} are the expected number of events for treatment arm 1 in the first and second stage, respectively. We also assume that the effect of one treatment does not affect the effect of other treatments in the study. Thus, in the variance–covariance matrix, the correlation is 0 for all entries corresponding to two different arms. For a (3:1) drop-the-losers, the variance–covariance matrix is defined as:

15391612, 2022, 1, Downloaded from https://onlinelibrary.wiley.com/doi/10.1002/pst.2168 by ${individualUser.givenNames} ${individualUser.surname} - University of Cambridge , Wiley Online Library on [15/11/2022]. See the Terms and Conditions (https://onlinelibrary.wiley.com/terms-and-conditions) on Wiley Online Library for rules of use; OA articles are governed by the applicable Creative Commons License

$$\Sigma = \begin{vmatrix} 1 & \rho_1 & 0 & 0 & 0 & 0 \\ \rho_1 & 1 & 0 & 0 & 0 & 0 \\ 0 & 0 & 1 & \rho_2 & 0 & 0 \\ 0 & 0 & \rho_2 & 1 & 0 & 0 \\ 0 & 0 & 0 & 0 & 1 & \rho_3 \\ 0 & 0 & 0 & 0 & \rho_3 & 1 \end{vmatrix}$$

The probability of rejecting a null hypothesis can be written as the sum of tail probabilities of multivariate normal distributions as:

$$P(reject\, H_{0k}) = P(\gamma_k = 1, Z_{2k} > c)$$

The conditions for this event can be represented as linear transforms of Z. The interim analysis can be described as a set of inequalities involving pairs of statistic tests, Z_{1k} and $Z_{1k'}$; and the final analysis involving the selected arm compared to the control, Z_{2k}. This set of inequalities considering all pairwise contrasts for each treatment arms are written in a contrast matrix, noted A. For the (3:1) drop-the-losers design ($K = 3$ and $J = 2$), the multivariate vector Z of test statistics matches to the columns of the contrast matrix A, that is, the first column of A describes inequalities involving Z_{11}, the second column Z_{21}, and so on. Under the configuration that the treatment 1 is selected for the final analysis and the rank of treatments is completely specified (i.e., $\gamma_1 = 1, \gamma_2 = 2, \gamma_3 = 3$), the contrast matrix A may be written as:

$$A = \begin{vmatrix} 1 & 0 & -1 & 0 & 0 & 0 \\ 0 & 0 & 1 & 0 & -1 & 0 \\ 0 & 1 & 0 & 0 & 0 & 0 \end{vmatrix}$$

The rows of this matrix A correspond to the following pairwise inequalities on test statistics:

- first row, comparison of treatment 1 versus treatment 2 at stage 1: $Z_{11} - Z_{12} > 0$
- second row, comparison of treatment 2 versus treatment 3 at stage 1: $Z_{12} - Z_{13} > 0$
- last row, comparison of treatment 1 versus the historical control at stage 2 with the decision rule based on the critical value, c: $Z_{21} - c > 0$

This matrix A is sufficient to describe the complete ranking of treatments as from the first and second contrast, we deduce the test statistic $Z_{11} - Z_{13} > 0$.

The multivariate normal vector of test statistics Z transformed with the matrix A is normal with mean $A\mu$ and covariance $A\Sigma A^T$. In this way, the probability of rejecting a hypothesis can be efficiently evaluated as the sum of tail probabilities of multivariate normal distributions. The probability of recommending treatment k, that is, rejecting H_{0k}, can be written as

$$P(reject\, H_{0k}|H_G) = P(\gamma_k = 1, Z_{2k} > c|H_G)$$

If we consider the probability to recommend a particular treatment k using complete rankings, there are $(K-1)!$ possible rankings. For instance, the two following rankings are applicable in order to recommend treatment 1 in a (3:1) drop-the-losers: $(\gamma_1 = 1, \gamma_2 = 2, \gamma_3 = 3)$ and $(\gamma_1 = 1, \gamma_2 = 3, \gamma_3 = 2)$. Under the global null hypothesis H_G, the probability of rejecting each of the null hypotheses, H_{0k}, is the same (in particular whatever the ranking considered). Without loss of generality, the probability to recommend a treatment arm $k = \{1, ..., K\}$ under H_G is given by:

$$P(reject\, H_{0k}|H_G) = (K-1)! \times P(\gamma_k = 1, ..., \gamma_{k'} = K, Z_{2k} > c, k \neq k'|H_G) \tag{3}$$

15391612, 2022, 1, Downloaded from https://onlinelibrary.wiley.com/doi/10.1002/pst.2168 by ${individualUser.givenNames} ${individualUser.surname} - University of Cambridge , Wiley Online Library on [15/11/2022]. See the Terms and Conditions (https://onlinelibrary.wiley.com/terms-and-conditions) on Wiley Online Library for rules of use; OA articles are governed by the applicable Creative Commons License

To complete our design, we need to compute the critical value c in order to get the desired FWER. Following the method specified above, the probability of interest in equation (3) is the tail probability of a multivariate normal distribution. The desired critical value, c, is found by solving equation (4) using the method of Genz and Bretz.[28]

$$P(\textit{reject at least one}\, H_{0k}|H_G) = K! \times \left(\int_0^{+\infty} \dots \int_c^{+\infty} f_{AZ}(x_1, \dots, x_K) dx_1, \dots, dx_K \right) \tag{4}$$

where f_{AZ} is the density function of a multivariate normal distribution $(A\mu, A\Sigma A^T)$. The critical value, c, is completely defined at the design stage. Under the global null hypothesis, H_G, the distribution of f_{AZ} is defined by both the design parameters and the correlation, ρ, which is defined by the expected numbers of events as shown above.

The above procedure for selection of a treatment arm at the interim analysis allows controlling the FWER in the strong sense. Of note, the contrasts matrix can be defined according to a partial ranking because the ranking of less promising arms is of no importance. For a (3:1) drop-the-losers, following the same example as above, the partial ordering ($\gamma_1 > \gamma_2$ and $\gamma_1 > \gamma_3$) suffices to specify the design. In this case, an alternative contrast matrix based on partial orderings, A^*, is:

$$A^* = \begin{vmatrix} 1 & 0 & -1 & 0 & 0 & 0 \\ 1 & 0 & 0 & 0 & -1 & 0 \\ 0 & 1 & 0 & 0 & 0 & 0 \end{vmatrix}$$

The contrasts Z_{11}-$Z_{12} > 0$ (first row) and Z_{11}-$Z_{13} > 0$ (second row) are specified. When a contrast matrix A* based on partial orderings is used, the sum of tail probabilities of multivariate normal distributions in Equations (3) and (4) must be multiplied by $(K-1)$ and K, respectively. Both methods gave similar critical values.

In a multi-arm setting the concept of power is complex due to the multiplicity of hypotheses. The probability to reject at least one false null hypothesis is called the disjunctive power, while the probability to reject all false null hypotheses is called the conjunctive power. Our formulation of the drop-the-losers design does not allow rejecting more than one null hypothesis, so the conjunctive power is not applicable. The disjunctive power constraints can be expressed conditioning on the number of effective treatment arms, L, $(L \leq K)$, expected under the alternative hypothesis H_1^L with φ representing the set of the L effective treatments and other treatments, belong to the complementary of φ, a null effect, $\delta_k \geq 1$. Let us first consider the probability of recommending a treatment arm $l \in \varphi$, which is:

$$P(\textit{reject}\, H_{0l}|H_1^{\varphi}) = P(\gamma_l = 1, Z_{2l} > c|H_1^{\varphi}), \text{ with } l \in \varphi \tag{5}$$

To get the probability in (5), we need to know the distribution of the test statistics under H_1^L and we must define the contrast matrix A* (with a partial ordering) that specify the recommendation of the treatment arm, l. Under the alternative, as opposed to the global null, each of the L treatment arms with an interesting effect can have a different distribution, noted $f^l{}_{A^*Z}$, depending on the effect size. For a given alternative hypothesis H_1^{φ}, the disjunctive power is the sum of L probabilities of recommending a treatment arm $l \in \varphi$ with an interesting effect, as follows:

$$P = P(\textit{reject at least one}\, H_{0l}|H_1^{\varphi}), \text{ with } l \in \varphi$$

$$P = \sum_{l \in \varphi} P(\gamma_l = 1, Z_{2l} > c|H_1^{\varphi})$$

$$P = \sum_{l \in \varphi} \left(\int_0^{+\infty} \dots \int_c^{+\infty} f^l{}_{A^*Z}(x_1, \dots, x_K) dx_1, \dots, dx_K \right) \tag{6}$$

where $f^l{}_{A^*Z}$ is the density function of a multivariate normal distribution under H_1^{φ} with a partial order contrast matrix that specifies the selection of the treatment arm l.

Based on the FWER and power constraint equations, we proposed the following algorithm to solve the critical value and the power of a (3:1) drop-the-losers design:

15391612, 2022, 1, Downloaded from https://onlinelibrary.wiley.com/doi/10.1002/pst.2168 by ${individualUser.givenNames} ${individualUser.surname} - University of Cambridge , Wiley Online Library on [15/11/2022]. See the Terms and Conditions (https://onlinelibrary.wiley.com/terms-and-conditions) on Wiley Online Library for rules of use; OA articles are governed by the applicable Creative Commons License

First step: the specification of the design (number of stages and number of treatment arms at each stage), the sample size at each stage and the nominal FWER allow to solve the critical value, c, for the end of the trial, using the constrain Equation (4) under the global null hypothesis.

Second step: the specification of the alternative scenario with one or more efficacious treatment arms allows one to get the distribution of the transformed test statistics, $f^l{}_{A^*Z}$.

Once the critical value c and the distributions under the alternative $f^l{}_{A^*Z}$ are known, the disjunctive power for a specific alternative hypothesis can be solved using the constraint Equation (6).

2.2 | The one-sample log-rank test

In order to extend the drop-the-losers design for time-to-event outcome, the one-sample log-rank test statistic, Z_{jk}, is used for each comparison of experimental arms k at stage j with the historical control data. First introduced by Breslow,[24] the one-sample log-rank test has been applied in single-arm phase II clinical trials designs.[22]

Let us assume n patients are recruited with individual failure times, T_i, and individual censoring times, C_i, for $i=\{1,...,n\}$. Then the observed time-to-event outcome and indicator for the ith patient are $X_i = min(T_i, C_i)$ and $\Delta_i = I(T_i \leq C_i)$, respectively. We assume that T_i and C_i are independent and identically distributed. The one-sample log-rank test is defined as:

$$Z = \frac{E-O}{\sqrt{E}} \tag{7}$$

where $O = \sum_1^n \Delta_i$ represents the observed number of events and $E = \sum_{i=1}^n \Lambda_0(X_i)$ the expected number of events (asymptotically). This number E is defined from $\Lambda_0(t)$ the cumulative hazard function of the historical control applied to the n observed time-to-events. $\Lambda_0(t)$ can be defined according to different parametric survival distributions such as exponential, Weibull, lognormal and gamma fitted on the historical control data. The sequential one-sample log-rank test uses only the data up to time t from the experimental group and all the data from the historical control group. Thus it must be defined from the treatment group. While the common two-sample log-rank score is the observed minus the expected numbers of events, the one-sample log-rank test score uses expected minus observed number of events to make its sign the same.[29] The one-sample log-rank statistic, Z, follows asymptotically a standard normal distribution under H_0 as shown by Wu.[30] However, this statistical test showed conservativeness in small samples.[21,30] Wu et al.[31] proposed a modified one-sample log-rank test defined as:

$$Z = \frac{E-\mathrm{O}}{\sqrt{\frac{\mathrm{O+E}}{2}}} \tag{8}$$

where O and E are the number of events in the experimental arm observed and expected, respectively. This test is also asymptotically standard normal distributed under the null hypothesis. Hence, we reject H_0 with one-sided type I error α, if $Z < Z_{1-\alpha}$, where $Z_{1-\alpha}$ is the $100(1-\alpha)$ percentile of the standard normal distribution. Wu[32] derived the distribution of the test under H_1, it is approximately normal with mean, μ, and unit variance. The use of the probability of failure under H_0 would underestimate the mean of the test statistic. The mean was computed using the average probabilities of failure under the null and the alternative.

$$\mathrm{Z} \sim N(\mu, 1)$$
$$\text{with } \mu = -\log(\delta) \times \sqrt{\frac{E_{H_0} + E_{H_1}}{2}}$$

where δ is the HR and where E_{H_0} and E_{H_1} are, respectively, the expected number of events under H_0 and H_1. If we extend the one-sample log-rank test to a multivariate normal distribution, then we get the following definition:

15391612, 2022, 1, Downloaded from https://onlinelibrary.wiley.com/doi/10.1002/pst.2168 by ${individualUser.givenNames} ${individualUser.surname} - University of Cambridge , Wiley Online Library on [15/11/2022]. See the Terms and Conditions (https://onlinelibrary.wiley.com/terms-and-conditions) on Wiley Online Library for rules of use; OA articles are governed by the applicable Creative Commons License

$$ZM\tilde{V}N\left\{\begin{pmatrix}-\log(\delta_1)\times\sqrt{E_{11}}\\-\log(\delta_1)\times\sqrt{E_{21}}\\-\log(\delta_2)\times\sqrt{E_{12}}\\-\log(\delta_2)\times\sqrt{E_{22}}\\-\log(\delta_3)\times\sqrt{E_{13}}\\-\log(\delta_3)\times\sqrt{E_{23}}\end{pmatrix},\begin{pmatrix}1&\rho_1&0&0&0&0\\\rho_1&1&0&0&0&0\\0&0&1&\rho_2&0&0\\0&0&\rho_2&1&0&0\\0&0&0&0&1&\rho_3\\0&0&0&0&\rho_3&1\end{pmatrix}\right\}$$

where E_{jk} is defined as the mean of the expected number of events under H_0 and H_1 at the stage j for the arm k.

Wu[31] showed that the modified one-sample log-rank test compares favorably to the 'classical' one-sample log-rank test[25] with respect to type I error rate control and power. Thus, the modified one-sample log-rank test will be used in the rest of the paper.

Figure 1 illustrates our proposal of a two-stage drop-the-losers (3:1) design. Patients are initially randomized in one of the three experimental arms (noted A, B and C). In this example, two experimental (B and C) arms are closed after the interim analysis based on the ranking of their test statistics. The treatment arm A is selected for the second stage. The final analysis uses a critical value, c, as a significance threshold for the final test statistic.

The estimation of the expected number of events in the three experimental arms will use the historical control of 265 relapsing osteosarcoma patients enrolled in the randomized multicenter open-label phase 3 trial.[33] They received high-dose methotrexate based chemotherapy or doxorubicin, ifosfamide and cisplatin based chemotherapy. The primary endpoint was the PFS and 189 events were observed. Figure 2 reports the non-parametric Kaplan–Meier PFS and two parametric estimations from Weibull and Generalized Gamma distributions. The Generalized Gamma regression model fits better with a lower AIC compared to the Weibull regression model.

3 | SIMULATION STUDY

We simulated a two-stage (3:1) drop-the-losers design using historical control, i.e. three randomized experimental arms with two stages and dropping the two least promising experimental arms after the first stage. The interim analysis takes place once the last first stage patient is recruited (which defined the administrative censoring date for the first stage analysis). We used the same sample size n_1 for all arms at the first stage and a potentially different sample size n_2 for the second stage. For comparison purposes, we also simulated three independent single arm trials with no interim analysis and called this approach the fixed design, as it is a non-adaptive design.

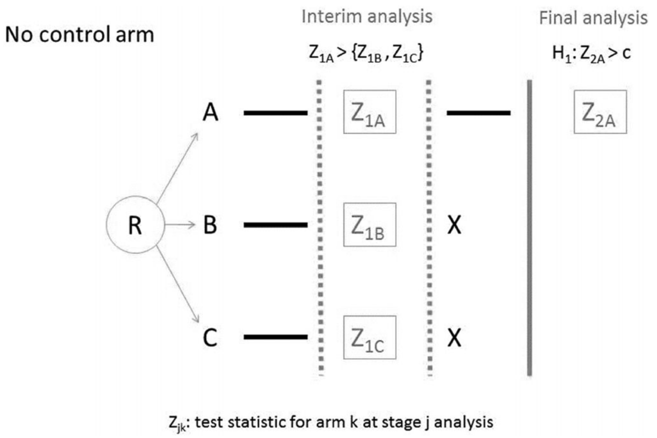

FIGURE 1 Overall outline of the two-stage drop-the-losers (3:1) design. The three experimental arms are noted A, B and C. The R stands for randomization and the X for the closure of experimental arms at interim analysis

15391612, 2022, 1, Downloaded from https://onlinelibrary.wiley.com/doi/10.1002/pst.2168 by ${individualUser.givenNames} ${individualUser.surname} - University of Cambridge , Wiley Online Library on [15/11/2022]. See the Terms and Conditions (https://onlinelibrary.wiley.com/terms-and-conditions) on Wiley Online Library for rules of use; OA articles are governed by the applicable Creative Commons License

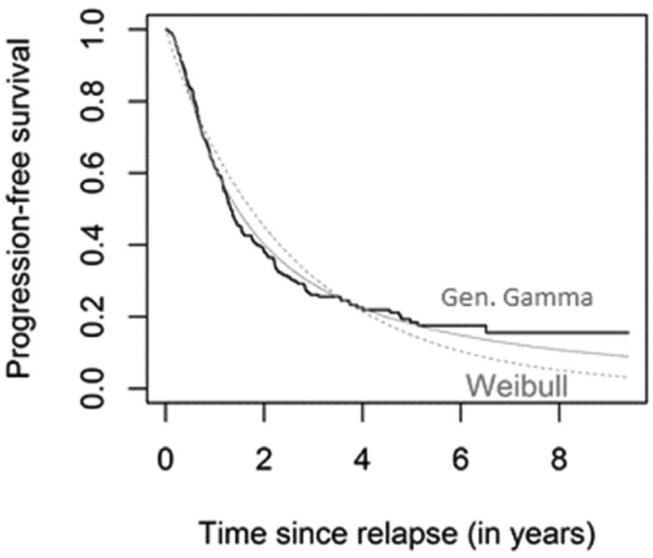

FIGURE 2 Kaplan–Meier estimate of progression-free survival of the historical control arm (in bold line; n = 265, 189 events) together with fitted parametric survival functions, Weibull (dotted line) and Generalized Gamma (solid line). The estimated parameters of the Weibull distribution were $\mu = 0.458$, $\sigma = 0.9509$ and $Q = 1$ and of the Generalized Gamma distribution were $\mu = 0.2051$, $\sigma = 1.2371$ and $Q = -0.4005$. Akaike information criterion of the parametric models: Weibull AIC = 735 and Gamma AIC = 697

TABLE 1 Simulated scenarios (S1-S9) corresponding to different relative treatment effect sizes of three experimental arms (A, B and C) compared to the historical control arm. The effect size is defined by the HR

Scenarios	Arm A	Arm B	Arm C
S1	null	null	null
S2	small	null	null
S3	medium	null	null
S4	large	null	null
S5	small	small	null
S6	medium	small	null
S7	medium	medium	null
S8	large	medium	null
S9	large	large	null

Note: Effect sizes: large $HR = 0.47$ ($\lambda = 0.215$), medium $HR = 0.60$ ($\lambda = 0.272$), small $HR = 0.76$ ($\lambda = 0.347$) and null $HR = 1$ ($\lambda = 0.458$); Hazard rate of the historical control arm $\lambda_0 = 0.458$ (obtained by fitting an exponential model on historical data).

3.1 | Simulation parameters

Individual survival data for the three experimental arms noted A, B and C were simulated according to an exponential distribution of parameters λ_A, λ_B and λ_C following the scenarios of Table 1. In the historical control, the 2-year PFS was 40% ($\lambda_0 = 0.458$). The null, small, medium and large effect sizes correspond to an increase in the 2-year PFS in the treatment arm of 0% ($HR = 1, \lambda = 0.458$), 10% ($HR = 0.76$, $\lambda = 0.347$), 18% ($HR = 0.60$, $\lambda = 0.272$) and 25% ($HR = 0.47$, $\lambda = 0.215$), respectively. These different effect sizes were attributed to one or more than one experimental arm among A, B and C resulting in 9 scenarios (Table 1). Scenario 1 represents the situation where no experimental arm is superior to the historical control arm. This corresponds to the global null hypothesis. Scenarios 2–4 include one non-null effect size. Scenarios 5–9 include two non-null effect sizes. At the second stage, the individual survival data of the selected treatment arm for n_2 patients were simulated using the same hazard rate than that used in the first stage. Censoring times were simulated using an exponential distribution ($\lambda = 0.5$). For the second stage analysis, the censoring administrative date was defined as the date when the last patient included reaches 2 years of follow-up. A complete

15391612, 2022, 1, Downloaded from https://onlinelibrary.wiley.com/doi/10.1002/pst.2168 by ${individualUser.givenNames} ${individualUser.surname} - University of Cambridge , Wiley Online Library on [15/11/2022]. See the Terms and Conditions (https://onlinelibrary.wiley.com/terms-and-conditions) on Wiley Online Library for rules of use; OA articles are governed by the applicable Creative Commons License

follow-up was assumed in all simulations. In the fixed design, parameters and exponential distributions were similar than in the drop-the-losers design.

3.2 | Design parameters

The three experimental arms were randomized with a balanced ratio. For each scenario, the following total sample sizes were considered: 40, 60, 80, 100, 120, 140, 160 and 200. For the drop-the-losers design, the total sample size splits into three arms in the first stage and one arm in the second stage, which makes ($3 \times n_1 + n_2$.). For the fixed design the total sample size n is divided by 3 in each arm $(\frac{n}{3}, \frac{n}{3}, \frac{n}{3})$. For the drop-the-losers design, different ratios $r = \frac{n_1}{n_2}$ between the first and second stage sample sizes were considered from 0.2 to 2.1. For instance, a ratio $r = 0.5$ means that for a total sample size of $n = 100$ patients, we have $n_1 = 20$ patients in each arm at the first stage and $n_2 = 40$ patients at the second stage. A uniform accrual rate of 50 patients per year was used. The modified one-sample log-rank test as defined in (8) was used in both the drop-the-losers and the fixed designs to assess efficacy of each experimental treatment against the historical control. The exponential parametric survival distribution was chosen to define $\Lambda_0(t)$ fitted on the historical control data. From our motivating phase II clinical trial, we chose a classical phase II 0.1 FWER; we also reported results for a 0.05 FWER. Computed at the design stage, the critical value used in the drop-the-losers design for testing the selected treatment at the final analysis was $c = 1.65$ to control the FWER at 0.10 (and $c = 1.91$ to control the FWER at 0.05).

Considering that the individual historical data may not follow the same survival distribution as the experimental arm, we also evaluated the impact of misspecification of the distribution in the one-sample log-rank statistic test for the drop-the-losers design. We simulated individual survival times for the experimental arms according to a Generalized Gamma distribution while an exponential distribution was used to compute the expected number of events. The parameters of the Generalized Gamma distribution (see Appendix for a detailed description of parameters) were estimated from the analysis of the historical control data with the flexsurv R package. The estimated parameters were $\mu = 0.2051$, $\sigma = 1.2371$ and $Q = -0.4005$.

In the fixed design, two analysis strategies were used depending on the consideration of the multiple testing. The first did not take into account any correction for multiplicity, this approach mimics three independent clinical trials. The second used Hommel's closed test procedure[34] to correct for multiplicity.

3.3 | Operating characteristics

The empirical FWER was estimated using scenario 1 where the three experimental arms have a null effect (also called the global null hypothesis). For the fixed design, the FWER was estimated similarly as for a multi-arm trial, by considering a global null hypothesis across the three experimental arms. Our formulation of the drop-the-losers design does not allow rejecting more than one null hypothesis, so we report the disjunctive power for both the drop-the-losers and the fixed designs. For scenario with two effective treatments arms (scenarios 5–9), the empirical power was calculated as the probability to reject the false null hypothesis for any one of the two effective treatment arms.

We replicated 10,000 simulated datasets for each scenario and computed the operating characteristics. All simulations were performed using R (R Core Team, 2018) mvtnorm[35] and flexsurv packages. All codes used for the simulations are available from https://github.com/Oncostat/DTLHC or https://github.com/rachidabbas-hub/DTLHC.

4 | RESULTS

First, we report the operating characteristics of the two-stage drop-the-losers design when the two stages sample sizes n_1 and n_2 are equal (section 4.1) and when they differ (in section 4.2). Second, we report in section 4.3, results when the historical data was mis-specified in the one-sample log-rank statistic test. Third, we compare our results with the fixed design in section 4.4.

15391612, 2022, 1, Downloaded from https://onlinelibrary.wiley.com/doi/10.1002/pst.2168 by ${individualUser.givenNames} ${individualUser.surname} - University of Cambridge , Wiley Online Library on [15/11/2022]. See the Terms and Conditions (https://onlinelibrary.wiley.com/terms-and-conditions) on Wiley Online Library for rules of use; OA articles are governed by the applicable Creative Commons License

4.1 | Operating characteristics of the drop-the-losers design, when $n_1 = n_2$

Table 2 reports that the FWER was controlled with the drop-the-losers design at the nominal level of 0.10, whatever the total sample size. The empirical power increased with the total sample size for scenarios 2–9. In scenario 4, for instance, the power increased from 0.44 with 40 patients to 0.80 with 120 patients and to 0.94 with 200 patients. In scenarios with one effective treatment arm (scenarios 2–4), we noted that the empirical power increased with the treatment effect size. In trials simulated with 120 patients, the empirical power increased from 0.27 for a small (HR = 0.76) treatment effect size to 0.58 for a medium (HR = 0.60) effect size and to 0.80 for large (HR = 0.47) effect size. Comparing scenarios with two effective treatment arms, the empirical power increased with the number of effective treatment arms. Again with 120 patients, the empirical power increased from 0.58 for one medium treatment effect size to 0.81 for two medium treatment effect sizes (scenarios 3 and 7). Among scenarios with two effective treatment arms (scenarios 5–9), some included different effect size: a small and a medium effect size for the scenario 6 and a medium and a large effect size for the scenario 8. In these scenarios, the treatment arm with the largest effect size was more frequently selected, on average around more than twice often. Higher was the total sample size, more frequent was the selection of the largest treatment effect size: in scenario 6, selections of the largest treatment effect size were 1.5 times more frequent for 40 patients and 2.6 times more frequent for 200 patients (see Table 3).

TABLE 2 Family-wise error rate (FWER) and empirical power (theoretical power in brackets) over the simulated scenarios (S1–S9) of the two stage drop-the-losers (3:1) design according to total sample sizes[a] (*N*) from 40 to 200 patients for a nominal FWER of 0.1

				Power								
N	**n1**	**n2**	**FWER**	**One effective treatment arm**			**Two effective treatment arms**					
			S1	S2	S3	S4	S5	S6	S7	S8	S9
40	10	10	0.10	0.14 (0.12)	0.29 (0.26)	0.44 (0.40)	0.23 (0.23)	0.36 (0.34)	0.45 (0.43)	0.57 (0.54)	0.65 (0.63)
60	15	15	0.10	0.16 (0.16)	0.35 (0.36)	0.53 (0.54)	0.28 (0.29)	0.44 (0.44)	0.55 (0.56)	0.68 (0.68)	0.76 (0.77)
80	20	20	0.09	0.20 (0.20)	0.44 (0.45)	0.64 (0.66)	0.33 (0.35)	0.53 (0.54)	0.65 (0.67)	0.77 (0.79)	0.85 (0.87)
100	25	25	0.09	0.23 (0.24)	0.51 (0.54)	0.73 (0.75)	0.39 (0.40)	0.60 (0.62)	0.74 (0.76)	0.85 (0.86)	0.91 (0.92)
120	30	30	0.08	0.27 (0.28)	0.58 (0.61)	0.80 (0.81)	0.43 (0.45)	0.67 (0.69)	0.81 (0.82)	0.90 (0.91)	0.95 (0.96)
140	35	35	0.09	0.30 (0.32)	0.66 (0.68)	0.85 (0.86)	0.48 (0.50)	0.72 (0.75)	0.86 (0.87)	0.93 (0.94)	0.97 (0.97)
160	40	40	0.08	0.33 (0.35)	0.72 (0.73)	0.90 (0.90)	0.53 (0.55)	0.77 (0.80)	0.89 (0.91)	0.96 (0.96)	0.98 (0.98)
200	50	50	0.08	0.40 (0.43)	0.80 (0.82)	0.94 (0.95)	0.59 (0.63)	0.85 (0.87)	0.95 (0.95)	0.98 (0.98)	0.99 (0.99)

[a]*N*: total sample size $= 3 \times n1 + n2$ with $n1 = n2$; n1: sample size per arm in stage 1; n2: sample size of the selected experimental arm for stage 2.

TABLE 3 Selection ratio of the best treatment arm in scenarios 6 and 8 according to total sample sizes[a] (*N*) from 40 to 200 patients and for a nominal FWER of 0.10

N[a]	**n1**	**n2**	**Scenario 6**[b]	**Scenario 8**[c]
40	10	10	1.62	1.64
60	15	15	1.53	1.49
80	20	20	1.67	1.55
100	25	25	1.75	1.57
120	30	30	1.93	1.78
140	35	35	2.13	1.91
160	40	40	2.30	2.07
200	50	50	2.64	2.40

[a]*N*: total sample size $= 3 \times n1 + n2$ with $n1 = n2$; n1: sample size per arm in stage 1; n2: sample size of the selected experimental arm for stage 2.
[b]The scenario 6 features two treatment arms with, respectively, a medium ($HR = 0.60$) and a small ($HR = 0.76$) effect size.
[c]The scenario 8 features two treatment arms with, respectively, a large ($HR = 0.47$) and a medium ($HR = 0.60$) effect size.

15391612, 2022, 1, Downloaded from https://onlinelibrary.wiley.com/doi/10.1002/pst.2168 by ${individualUser.givenNames} ${individualUser.surname} - University of Cambridge , Wiley Online Library on [15/11/2022]. See the Terms and Conditions (https://onlinelibrary.wiley.com/terms-and-conditions) on Wiley Online Library for rules of use; OA articles are governed by the applicable Creative Commons License

The drop-the-losers design controlled as well the FWER at the nominal level of 0.05 and similar patterns of the empirical power across different sample sizes, number of effective arms, and different effect sizes were observed (Table 4). In these sets of simulations Tables 2 and 4, the theoretical power was very close to the empirical power estimates from simulations.

4.2 | Unequal sample sizes per arm between first and second stages, $n_1 \neq n_2$

Here, we report operating characteristics of the two-stage drop-the-losers design in cases where different sample size is used at the second stage. No impact of the r ratio was observed on the FWER. When more patients were accrued during the second stage ($r < 1$), the power tended to decrease when the effect size was medium or large (Figure 3). For instance, in scenario 4 with 120 patients, the power was 0.80 for a ratio of 1 and decreased to 0.75 with a ratio of 0.5. The impact of lowering the ratio, r, on the design's power was larger with larger sample sizes: with a sample size of 160 patients in scenario 4 the power decreased from 0.89 with a ratio of 1 (40 patients per arm in stage 1 and stage 2) to 0.70 with a ratio of 0.2 (20 patients per arm in stage 1 and 100 patients in stage 2). With sample sizes smaller than 100 patients, the impact of lowering the ratio was conversely to an increase in empirical power. For instance, in scenario 7 with 80 patients, the power was 0.65 and increased to 0.68 with a ratio of 0.5. For cases where more patients were accrued during the first stage ($r > 1$), no sensible change in power was observed in all the simulations. The r ratio showed little impact on the power in cases where the effect size was small (scenarios 2 and 5).

Similar patterns in terms of FWER and empirical power across different ratios were observed for a nominal FWER of 0.05 (Figure 4). The same decrease in power was observed in scenarios 4 and 3 which feature large and medium effect sizes. In scenario 4 with 140 patients, the power was 0.84 for a ratio of 1 and decreased to 0.80 with a ratio of 0.5.

4.3 | Impact of misspecification of the survival distribution of the historical data

In the case of misspecification of the survival distribution of the historical data, the FWER inflated whatever the sample size (Table 5). The FWER increased with higher sample sizes in case of misspecification (from 0.11 to 0.24 with 40 and 200 patients, respectively) whereas it tended to decrease in case of correct specification (from 0.10 to 0.08 with 40 and 200 patients, respectively). The source of the FWER inflation was an overestimation of the one-sample log-rank test score. In scenario 1 with 80 patients, the mean the one-sample log-rank test score was 0.33 under misspecification versus 0.10 under correct specification. The higher was the sample size, the higher the empirical power; the higher was the treatment effect size, the higher the empirical power (see Table 5 and Figure 5). For instance, in scenarios 2–4 with one effective treatment arm, the empirical power of trials with 120 patients was 0.45 for a small (HR = 0.76) treatment effect size and increased to 0.81 and 0.93 with medium (HR = 0.60) and large (HR = 0.47) treatment effect size,

TABLE 4 Family-wise error rate (FWER) and empirical power (theoretical power in brackets) over the simulated scenarios (S1-S9) of the two stage drop-the-losers (3:1) design according to total sample sizes[a] (N) from 40 to 200 patients for a nominal FWER of 0.05

				Power								
N	n1	n2	FWER	One effective treatment arm			Two effective treatment arms					
			S1	S2	S3	S4	S5	S6	S7	S8	S9
40	10	10	0.05	0.09 (0.08)	0.22 (0.19)	0.36 (0.33)	0.16 (0.14)	0.27 (0.24)	0.33 (0.32)	0.45 (0.43)	0.53 (0.52)
60	15	15	0.05	0.11 (0.11)	0.28 (0.28)	0.48 (0.48)	0.19 (0.19)	0.34 (0.33)	0.45 (0.45)	0.58 (0.59)	0.68 (0.69)
80	20	20	0.05	0.13 (0.14)	0.36 (0.37)	0.60 (0.61)	0.23 (0.24)	0.42 (0.43)	0.55 (0.56)	0.70 (0.71)	0.80 (0.81)
100	25	25	0.04	0.16 (0.17)	0.45 (0.46)	0.71 (0.71)	0.28 (0.29)	0.50 (0.51)	0.64 (0.66)	0.79 (0.81)	0.88 (0.89)
120	30	30	0.04	0.19 (0.2)	0.53 (0.54)	0.78 (0.79)	0.31 (0.33)	0.57 (0.59)	0.72 (0.74)	0.85 (0.87)	0.93 (0.94)
140	35	35	0.05	0.22 (0.23)	0.59 (0.61)	0.84 (0.85)	0.35 (0.38)	0.63 (0.66)	0.79 (0.81)	0.90 (0.92)	0.96 (0.97)
160	40	40	0.05	0.25 (0.27)	0.65 (0.68)	0.88 (0.89)	0.41 (0.43)	0.69 (0.72)	0.84 (0.86)	0.94 (0.95)	0.98 (0.98)
200	50	50	0.04	0.30 (0.33)	0.76 (0.78)	0.94 (0.94)	0.47 (0.51)	0.78 (0.81)	0.91 (0.93)	0.97 (0.98)	0.99 (0.99)

[a]N: total sample size $= 3 \times n1 + n2$ with $n1 = n2$; n1: sample size per arm in stage 1; n2: sample size of the selected experimental arm for stage 2.

15391612, 2022, 1, Downloaded from https://onlinelibrary.wiley.com/doi/10.1002/pst.2168 by ${individualUser.givenNames} ${individualUser.surname} - University of Cambridge , Wiley Online Library on [15/11/2022]. See the Terms and Conditions (https://onlinelibrary.wiley.com/terms-and-conditions) on Wiley Online Library for rules of use; OA articles are governed by the applicable Creative Commons License

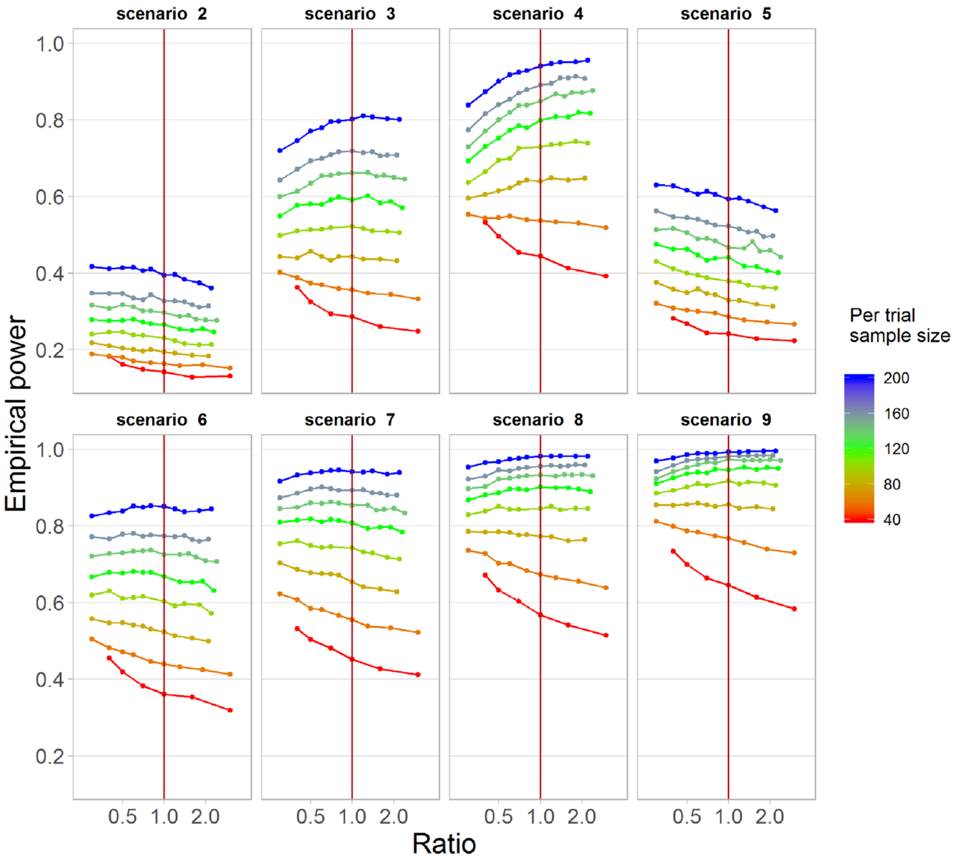

FIGURE 3 The empirical power of the two-stage drop-the-losers (3:1) design with historical control arm according to the ratio $r = n_1/n_2$ for each scenario and each trial sample size (from 40 to 200) for a nominal FWER of 0.1. The horizontal axis scale is the base-2 logarithm of the ratio r. The vertical red line marks designs where $r = 1$ (See Table 2 for numerical results)

respectively. The same pattern was observed for scenarios 5–9 with two effective treatment arms. In scenarios 6 and 8, the largest effect size treatment arm was the most frequently selected. Misspecification led to higher empirical power, up to 0.28 difference, as compared with the correct specification case (0.87 vs. 0.59 in scenario 5 with 200 patients). The largest differences were observed with small treatment effect size and a large number of patients. The source of the empirical power inflation was an overestimation of the one-sample log-rank test score. For instance, under misspecification in scenario 4, the mean one-sample log-rank test score was 3.54 versus 2.37 under correct specification.

Similar patterns of the FWER and power related to misspecification were observed for a nominal FWER of 0.05 (Table 6). The FWER was inflated from 0.06 with 40 patients up to 0.14 with 200 patients. The empirical power was 0.77 in scenario 3 with 120 patients. The empirical power also increased under misspecification (Figure 6).

In case of misspecification, the theoretical power was much lower than the empirical power estimates from simulations, the highest differences were observed for small sample sizes and scenarios including large treatment effect sizes (Tables 5 and 6).

4.4 | Comparison of the two-stage drop-the-losers design with the fixed design

The nominal 0.10 FWER which was controlled in the drop-the-losers design was inflated in the fixed design not corrected for multiplicity. The smaller was the sample size, the higher FWER inflation: from 0.22 FWER with the largest 200 sample size to 0.29 FWER with the smallest 40 patients sample size. After correction for multiplicity, the nominal 0.10 FWER was slightly inflated in the fixed design for sample sizes below 80 patients: 0.11 FWER for 60 patients and 0.12 FWER for 40 patients. The fixed design showed higher empirical power than the drop-the-losers design. The average power of uncorrected fixed design over scenarios 2–9 whatever the sample size was 0.15 higher than the one of the drop-the-losers design. For instance, the powers in scenario 7 with 120 patients were 0.94 for the fixed design and

15391612, 2022, 1, Downloaded from https://onlinelibrary.wiley.com/doi/10.1002/pst.2168 by ${individualUser.givenNames} ${individualUser.surname} - University of Cambridge , Wiley Online Library on [15/11/2022]. See the Terms and Conditions (https://onlinelibrary.wiley.com/terms-and-conditions) on Wiley Online Library for rules of use; OA articles are governed by the applicable Creative Commons License

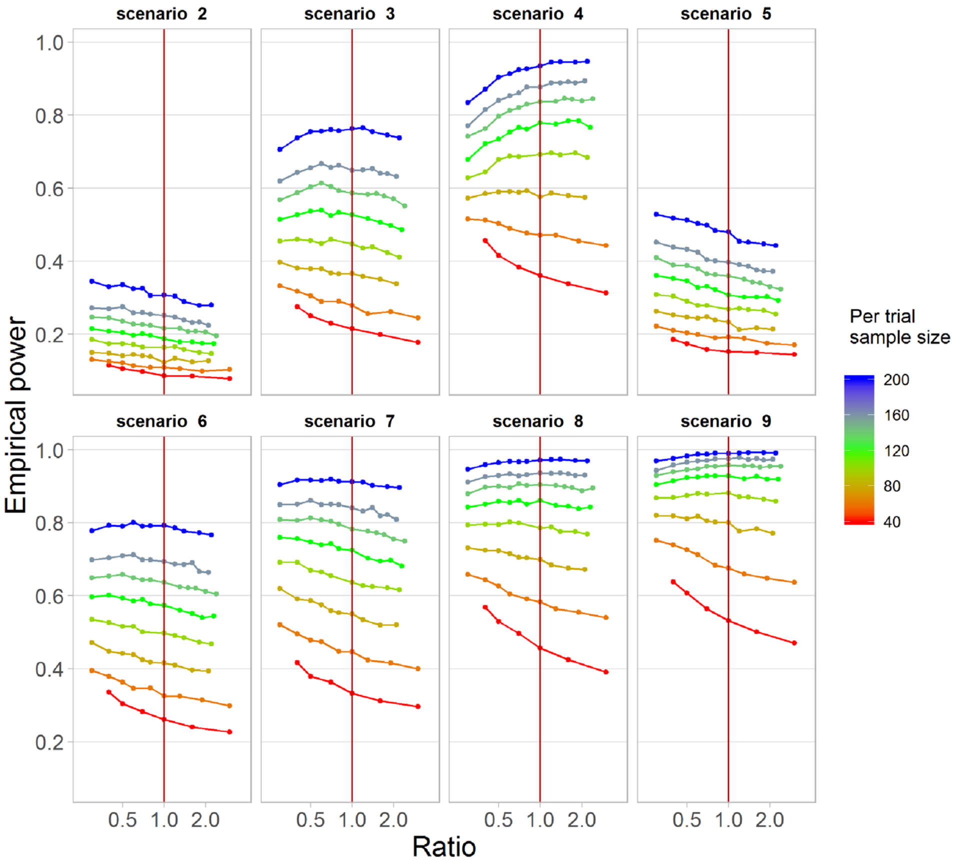

FIGURE 4 The empirical power of the two-stage drop-the-losers (3:1) design with historical control arm according to the ratio $r = n_1/n_2$ for each scenario and each trial sample size (from 40 to 200) for a nominal FWER of 0.05. The horizontal axis scale is the base-2 logarithm of the ratio r. The vertical red line marks designs where $r = 1$ (See Table 3 for numerical results)

0.81 with the drop-the-losers design. After correction for multiplicity, the powers were similar in fixed design and the drop-the-losers design (See Table A.1 in Appendix).

Similar inflation of the FWER in the fixed design was observed with a nominal 0.05 FWER. The empirical power of the fixed design after correction and of the drop-the-losers design were also comparable (See Table A.2 in Appendix).

5 | DISCUSSION

In this paper, the drop-the-losers design has been extended enabling simultaneous assessment for efficacy of several experimental treatments compared to a historical control for a time-to-event outcome. We proposed an algorithm to solve the sample size based on a set of parameters at the design stage. Overall, the procedure controlled the FWER provided that the survival of the historical control arm is correctly specified. The theoretical power based on the proposed algorithm matched well the empirical power estimates from simulations. The empirical power increased with the total sample size, the treatment effect size, and the number of effective treatment arms. In cases with two effective treatment arms, the drop-the-losers design was able to pick the treatment with the largest effect size. The simulation study provided a broad range of settings to determine the influence of several design specifications on the operational characteristics of the design. First, the ratio of the first stage over the second stage sample size per arm was key to preserve the power of the trial. We observed that including more patients per arm at the second stage than at the first, resulted in a loss of power. Reducing the first stage sample size may reduce the probability to select the best treatment arm. Conversely, with many patients at the first stage, the interim analysis may come too late and the design loses interest. The choice of a time (and the number of patients) for the interim analysis is complex. Second, the misspecification of the survival distribution of historical control inflated both the FWER and the empirical power. A bias was observed in the expected number of events under misspecification, consistently

15391612, 2022, 1, Downloaded from https://onlinelibrary.wiley.com/doi/10.1002/pst.2168 by ${individualUser.givenNames} ${individualUser.surname} - University of Cambridge , Wiley Online Library on [15/11/2022]. See the Terms and Conditions (https://onlinelibrary.wiley.com/terms-and-conditions) on Wiley Online Library for rules of use; OA articles are governed by the applicable Creative Commons License

TABLE 5 Family-wise error rate (FWER) and empirical power (theoretical power in brackets) over the simulated scenarios (S1–S9) of the two stage drop-the-losers (3:1) design with misspecification of historical data according to total sample sizes[a] (N) from 40 to 200 patients for a nominal FWER of 0.1. Historical control group is mis-specified in the sense that an exponential survival model was used in the one-sample log rank test while survival data of the experimental arms were simulated according to a generalized gamma distribution

				Power								
N	n1	n2	FWER	One effective treatment arm			Two effective treatment arms					
			S1	S2	S3	S4	S5	S6	S7	S8	S9
40	10	10	0.11	0.24 (0.15)	0.49 (0.28)	0.70 (0.39)	0.35 (0.27)	0.54 (0.38)	0.63 (0.48)	0.79 (0.56)	0.83 (0.63)
60	15	15	0.13	0.27 (0.20)	0.55 (0.40)	0.76 (0.54)	0.41 (0.35)	0.63 (0.50)	0.75 (0.62)	0.86 (0.71)	0.91 (0.78)
80	20	20	0.13	0.32 (0.26)	0.66 (0.50)	0.84 (0.66)	0.50 (0.43)	0.72 (0.61)	0.84 (0.74)	0.91 (0.82)	0.96 (0.88)
100	25	25	0.15	0.39 (0.31)	0.73 (0.60)	0.89 (0.75)	0.58 (0.50)	0.80 (0.70)	0.9 (0.82)	0.95 (0.89)	0.98 (0.93)
120	30	30	0.16	0.45 (0.37)	0.81 (0.68)	0.93 (0.82)	0.67 (0.57)	0.87 (0.77)	0.94 (0.88)	0.97 (0.93)	0.99 (0.96)
140	35	35	0.18	0.52 (0.42)	0.85 (0.74)	0.95 (0.87)	0.73 (0.63)	0.90 (0.82)	0.96 (0.92)	0.98 (0.96)	0.99 (0.98)
160	40	40	0.19	0.57 (0.47)	0.88 (0.80)	0.97 (0.91)	0.78 (0.68)	0.93 (0.87)	0.98 (0.95)	0.99 (0.97)	1.00 (0.99)
200	50	50	0.24	0.66 (0.56)	0.93 (0.87)	0.99 (0.95)	0.87 (0.77)	0.97 (0.92)	0.99 (0.98)	1.00 (0.99)	1.00 (1.00)

[a] N: total sample size $= 3 \times n1 + n2$ with $n1 = n2$; n1: sample size per arm in stage 1; n2: sample size of the selected experimental arm for stage 2.

15391612, 2022, 1, Downloaded from https://onlinelibrary.wiley.com/doi/10.1002/pst.2168 by ${individualUser.givenNames} ${individualUser.surname} - University of Cambridge , Wiley Online Library on [15/11/2022]. See the Terms and Conditions (https://onlinelibrary.wiley.com/terms-and-conditions) on Wiley Online Library for rules of use; OA articles are governed by the applicable Creative Commons License

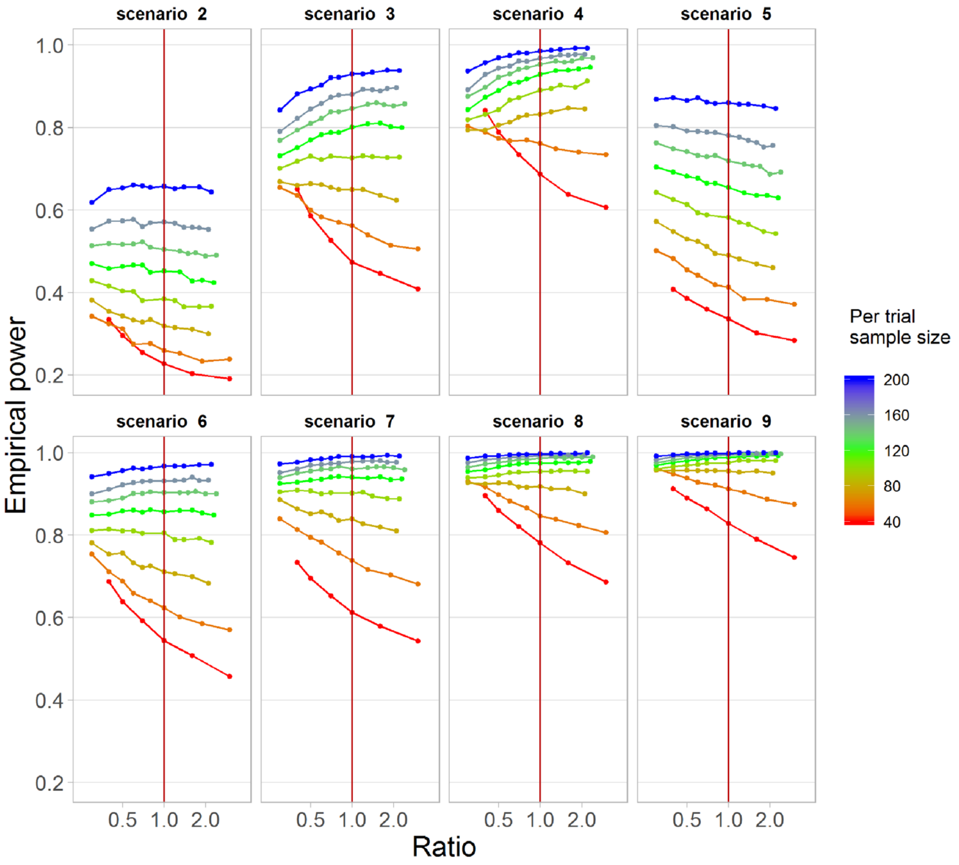

FIGURE 5 The empirical power of the two-stage drop-the-losers (3:1) design with mis-specified historical control arm according to the ratio $r = n_1/n_2$ for each scenario and each trial sample size (from 40 to 200) for a nominal FWER of 0.1. The horizontal axis scale is the base-2 logarithm of the ratio r. The vertical red line marks designs where $r = 1$ (see numerical results in Table 4). Historical control group is mis-specified in the sense that an exponential survival model was used in the one-sample log rank test while data were simulated according to a log-gamma distribution

TABLE 6 Family-wise error rate (FWER) and empirical power (theoretical power in brackets) over the simulated scenarios (S1–S9) of the two stage drop-the-losers (3:1) design with misspecification of historical data according to total sample sizes[a] (N) from 40 to 200 patients at stage 1 and stage 2 for a nominal FWER of 0.05. Historical control group is mis-specified in the sense that an exponential survival model was used in the one-sample log rank test while survival data of the experimental arms were simulated according to a generalized gamma distribution

				Power								
N	n1	n2	FWER	One effective treatment arm			Two effective treatment arms					
			S1	S2	S3	S4	S5	S6	S7	S8	S9
40	10	10	0.06	0.16 (0.10)	0.38 (0.21)	0.61 (0.32)	0.23 (0.18)	0.42 (0.28)	0.49 (0.36)	0.69 (0.45)	0.73 (0.53)
60	15	15	0.07	0.19 (0.14)	0.48 (0.32)	0.72 (0.48)	0.29 (0.24)	0.52 (0.40)	0.65 (0.52)	0.79 (0.63)	0.87 (0.71)
80	20	20	0.07	0.25 (0.19)	0.59 (0.44)	0.81 (0.62)	0.38 (0.31)	0.63 (0.51)	0.77 (0.65)	0.88 (0.76)	0.94 (0.84)
100	25	25	0.08	0.30 (0.23)	0.69 (0.54)	0.89 (0.73)	0.46 (0.38)	0.72 (0.61)	0.85 (0.75)	0.94 (0.85)	0.97 (0.91)
120	30	30	0.09	0.37 (0.28)	0.77 (0.63)	0.92 (0.81)	0.55 (0.45)	0.81 (0.69)	0.91 (0.83)	0.97 (0.91)	0.99 (0.95)
140	35	35	0.10	0.44 (0.33)	0.83 (0.70)	0.95 (0.86)	0.64 (0.51)	0.86 (0.76)	0.95 (0.88)	0.98 (0.94)	0.99 (0.97)
160	40	40	0.12	0.49 (0.38)	0.87 (0.77)	0.97 (0.90)	0.69 (0.57)	0.91 (0.81)	0.97 (0.92)	0.99 (0.97)	1.00 (0.98)
200	50	50	0.14	0.60 (0.47)	0.93 (0.86)	0.99 (0.95)	0.80 (0.67)	0.95 (0.89)	0.99 (0.97)	1.00 (0.99)	1.00 (0.99)

[a]N: total sample size $= 3 \times n1 + n2$ with $n1 = n2$; n1: sample size per arm in stage 1; n2: sample size of the selected experimental arm for stage 2.

15391612, 2022, 1, Downloaded from https://onlinelibrary.wiley.com/doi/10.1002/pst.2168 by ${individualUser.givenNames} ${individualUser.surname} - University of Cambridge , Wiley Online Library on [15/11/2022]. See the Terms and Conditions (https://onlinelibrary.wiley.com/terms-and-conditions) on Wiley Online Library for rules of use; OA articles are governed by the applicable Creative Commons License

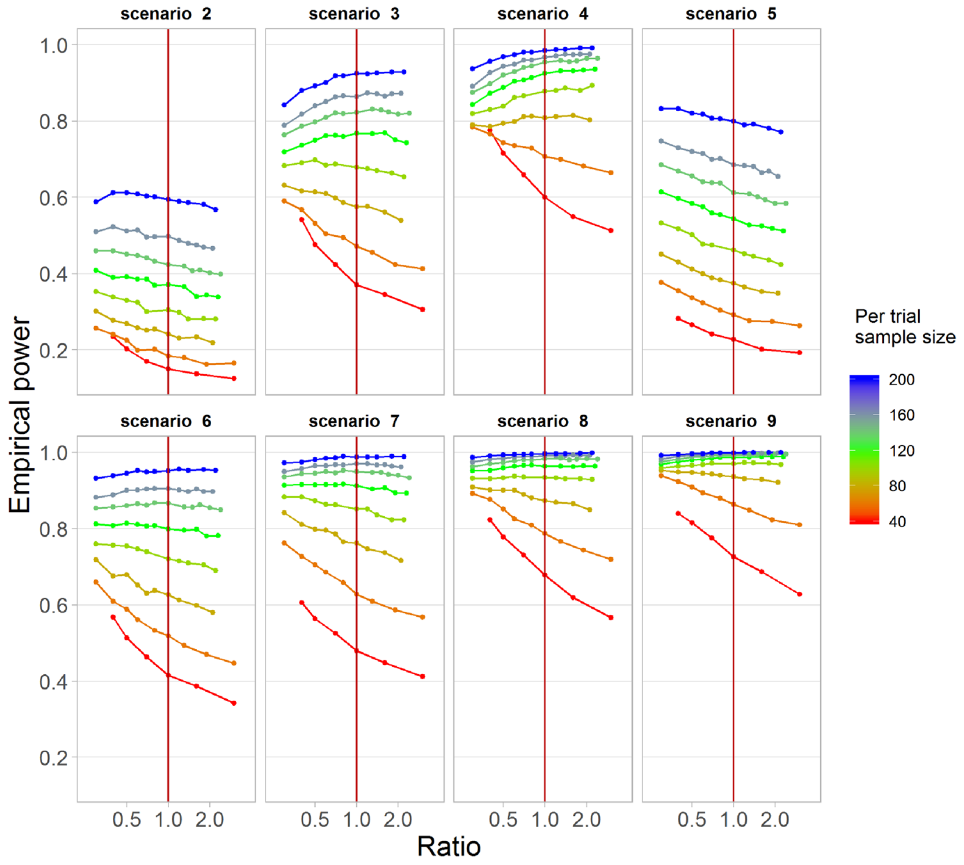

FIGURE 6 The empirical power of the two stage drop-the-losers (3:1) design with mis-specified historical control arm according to the ratio $r = n_1/n_2$ for each scenario and each trial sample size (from 40 to 200) for a nominal FWER of 0.05. The horizontal axis scale is the base-2 logarithm of the ratio r. The vertical red line marks designs where $r = 1$ (see numerical results in Table 5). Historical control group is mis-specified in the sense that an exponential survival model was used in the one-sample log rank test while data were simulated according to a log-gamma distribution

with a report from Wu et al.[23] The more patients were included, the greater the bias: this explains why the FWER and the empirical power increased with sample size. When compared with the correct specification, the greatest differences in FWER and empirical power were observed for large sample sizes and small treatment effects. Without correction for multiple testing, the fixed design approach doubled the FWER. Correcting for multiplicity did not completely solve the FWER inflation for small sample sizes.

Our design applies under several conditions, and it is usually applied with only one interim analysis. The one-sample log-rank test used in our design relies on the proportional hazards assumption. The application of our design needs to check this assumption. The impact of non-proportional hazards on the design should be evaluated in further research. An interesting solution might be the combination test based on the restricted mean survival time.[36] The historical control approach must be used with caution. This method assumes that historical data is sufficiently similar to the contemporaneous control patient population. Otherwise, point estimates may harbor bias, and the nominal type I error rate can be inflated. Usually, the historical individual patient data should come from previous clinical trials, ideally conducted in the same centers and sufficiently representative of the targeted population. Concerning the number of interim analyses, Kwak and Jung[22] said that no significant gain was associated with additional stages above two stages. Wason[6] suggested that only trials with more than four arms could benefit from an additional stage over two stages. In rare cancers setting, it is unlikely to screen more than four contemporaneous experimental arms with drugs at the comparable time of their development.

Applying our innovative design in rare and severe diseases deserves some practical recommendations. First, investigators of the trial must be aware of conditions for applying the drop-the-losers approach: proportional hazards assumption has to be checked and modeling of historical data carefully evaluated since a poorly fitted model can compromise the validity of the study. Both statisticians and investigators should discuss whether the drop-the-losers approaches are

15391612, 2022, 1, Downloaded from https://onlinelibrary.wiley.com/doi/10.1002/pst.2168 by ${individualUser.givenNames} ${individualUser.surname} - University of Cambridge , Wiley Online Library on [15/11/2022]. See the Terms and Conditions (https://onlinelibrary.wiley.com/terms-and-conditions) on Wiley Online Library for rules of use; OA articles are governed by the applicable Creative Commons License

pertinent in their clinical setting to take full advantage of these methods.[37] Our second recommendation is justifying the sample size according to an extensive simulation study. Given the setting of the trial motivating this study, a sample size of 120 patients (30 patients per experimental arms) seems the acceptable minimum (as far as a power of 80% is considered acceptable in phase II in rare diseases). Planning to include a greater proportion of patients in the second stage did not notably improve the power and even reduced it for larger sample sizes. As a rule of thumb, we recommend to use for the first stage the same or a higher number of patients than in the second stage. Thirdly, the follow-up time of patients included in interim analysis should be long enough for the detection of potential treatment effects but shorter than the time of recruiting remaining patients. Depending on the event rate, one can calculate the expected power of the interim analysis to judge if the timing of the interim analysis is appropriate. Thus, the optimal follow-up time is a trade-off which depends on the event rate and recruitment rate of the trial. Wason et al. showed an example of a phase II trial (Immunotace, ISRTCN:11889464) where the interim analysis was useless because at time of interim analysis – recruitment of stage 1 patients plus their follow-up time- the total number of patients had been already included.[37]

Limitations of our design include the selection of one and only one treatment for the second stage, even under the global null hypothesis. On one hand, when no experimental arm is effective (null hypothesis), a futility rule able to stop the trial at the interim analysis could be added. To our knowledge, such a futility rule does not exist in the drop-the-losers approaches but it can be evaluated in future research. In an application of this design, the best performing arm may be dropped at the interim due to safety reasons or other concerns. Wason et al. showed that the procedure described here allows for a maximal control of the FWER. Thus any deviation from this procedure which results in a lower number of arms, or lower ranked arms, progressing would result in a conservative design, that is, the FWER would be lower than the nominal level. On the other hand, our design is underpowered when multiple effective experimental arms are in the trial. In our motivating trial, since at most one arm is expected to be effective, we favored speedy recruitment rather than keeping the possibility of selecting several candidates. Our design could be extended for selecting several potentially effective arms in settings where several promising therapies are in development.

Our design has several advantages over traditional approaches. Our design aims sparing randomization to a contemporaneous control group when individual patient data from a suitable historical cohort are available. The adaptive 'drop-the-losers' rule favors the recruitment of patients in the most promising arm. In a trial of total sample size n, the fixed design recruits $\frac{n}{3}$ patients in the most effective arm, whereas the two-stage (3:1) drop-the-losers design with a ratio $r = 1$ recruits $\frac{n}{2}$ patients. Taking a trial with 120 patients as an example, 40 patients were exposed to the most effective treatment in the fixed regimen versus 60 patients in the two-stage (3:1) drop-the-losers design. The interim analysis is usually triggered based on the number of events observed. However, this strategy has a drawback in multi-arm trials: if one experimental treatment with poor efficacy leads to the observation of many events, most of the information at the time of the interim analysis would be accumulated in this poorly performing arm. The fraction of information for other arms would be too low to have powerful tests. We thus retained for the timing of analysis the time needed to recruit the pre-specified number of patients for the first stage plus the minimal required follow-up time for the last included first stage patient. Based on the number of patients included, our interim analysis can be done without the impact of poorly performing treatment arms on the others.

In conclusion, our proposal taking advantage of historical data and the multi-arm design can help to speed up the screening of treatments for rare diseases. Our design also has the advantage of using a time-to-event outcome which is very popular for treatment efficacy assessment in oncology clinical trials and we developed an algorithm to solve the sample size for the study design. Compared to a more classical approach based on multiple single-arm trials, our approach achieves a strict control of the FWER and includes more patients in the most promising arm. These features give to the proposed design advantages from both sponsor's, and patients' perspectives. For patients, the design gives a higher likelihood to receive a more promising treatment, which makes the trial more attractive to them – this should be the subject of collaborative patient-centered studies. For the sponsor, the trial is more attractive because patients' accrual is easier and the likelihood of detecting an effective treatment is increased as several treatments are assessed simultaneously and more patients are included in the most promising arm. The two-stage drop-the-losers design for time-to-event outcome using a historical control arm is a promising tool to meet the challenge of improving phase II clinical trials evaluating the many innovative treatments that are emerging in the immuno-oncology space.

CONFLICT OF INTEREST

The authors have declared no conflict of interest.

DATA AVAILABILITY STATEMENT

Data sharing is not applicable to this article as no new data were created or analyzed in this study.

15391612, 2022, 1, Downloaded from https://onlinelibrary.wiley.com/doi/10.1002/pst.2168 by ${individualUser.givenNames} ${individualUser.surname} - University of Cambridge , Wiley Online Library on [15/11/2022]. See the Terms and Conditions (https://onlinelibrary.wiley.com/terms-and-conditions) on Wiley Online Library for rules of use; OA articles are governed by the applicable Creative Commons License

ORCID

Rachid Abbas https://orcid.org/0000-0002-9737-4548
James Wason https://orcid.org/0000-0002-4691-126X
Stefan Michiels https://orcid.org/0000-0002-6963-2968
Gwénaël Le Teuff https://orcid.org/0000-0003-3292-0939

REFERENCES

1. Rosenberg SA. Entering the mainstream of cancer treatment. *Nat Rev Clin Oncol*. 2014;11(11):630-632. https://doi.org/10.1038/nrclinonc.2014.174
2. Bishop MW, Janeway KA, Gorlick R. Future directions in the treatment of osteosarcoma. *Curr Opin Pediatr*. 2016;28(1):26-33. https://doi.org/10.1097/MOP.0000000000000298
3. Kansara M, Teng MW, Smyth MJ, Thomas DM. Translational biology of osteosarcoma. *Nat Rev Cancer*. 2014;14(11):722-735. https://doi.org/10.1038/nrc3838
4. Gong J, Chehrazi-Raffle A, Reddi S, Salgia R. Development of PD-1 and PD-L1 inhibitors as a form of cancer immunotherapy: a comprehensive review of registration trials and future considerations. *J Immunother Cancer*. 2018;6(1):8. https://doi.org/10.1186/s40425-018-0316-z
5. Gorlick R, Janeway K, Lessnick S, Randall RL, Marina N. Children's oncology Group's 2013 blueprint for research: bone tumors. *Pediatr Blood Cancer*. 2013;60(6):1009-1015. https://doi.org/10.1002/pbc.24429
6. Wason JMS. Reducing the average number of patients needed in a phase II trial through novel design. *Clin Res Regul Aff*. 2013;30(4):47-54. https://doi.org/10.3109/10601333.2013.854802
7. Parmar MKB, Carpenter J, Sydes MR. More multiarm randomised trials of superiority are needed. *Lancet*. 2014;384(9940):283-284. https://doi.org/10.1016/S0140-6736(14)61122-3
8. Pocock SJ. Group sequential methods in the design and analysis of clinical trials. *Biometrika*. 1977;64(2):191-199. https://doi.org/10.1093/biomet/64.2.191
9. Bauer P. Multiple testing in clinical trials. *Stat Med*. 1991;10:871-890. https://doi.org/10.1002/sim.4780100609
10. Müller H-H, Schäfer H. Adaptive group sequential designs for clinical trials: combining the advantages of adaptive and of classical group sequential approaches. *Biometrics*. 2001;57(3):886-891. https://doi.org/10.1111/j.0006-341X.2001.00886.x
11. Brannath W, Koenig F, Bauer P. Multiplicity and flexibility in clinical trials. *Pharm Stat*. 2007;6(3):205-216. https://doi.org/10.1002/pst.302
12. Bauer P, Bretz F, Dragalin V, König F, Wassmer G. Twenty-five years of confirmatory adaptive designs: opportunities and pitfalls. *Stat Med*. 2016;35(3):325-347. https://doi.org/10.1002/sim.6472
13. Ghosh P, Liu L, Senchaudhuri P, Gao P, Mehta C. Design and monitoring of multi-arm multi-stage clinical trials. *Biometrics*. 2017;73(4):1289-1299. https://doi.org/10.1111/biom.12687
14. Pallmann P, Bedding AW, Choodari-Oskooei B, et al. Adaptive designs in clinical trials: why use them, and how to run and report them. *BMC Med*. 2018;16(1):29. https://doi.org/10.1186/s12916-018-1017-7
15. Food and Drug Administration (FDA). *Adaptive designs for clinical trials of drugs and biologics guidance for industry DRAFT*; 2018. https://www.fda.gov/Drugs/GuidanceComplianceRegulatoryInformation/Guidances/default.htm.
16. Thall PF, Simon R, Ellenberg SS. A two-stage Design for Choosing among several experimental treatments and a control in clinical trials. *Biometrics*. 1989;45(2):537-547. https://doi.org/10.2307/2531495
17. Sampson AR, Sill MW, Bauer P, et al. Drop-the-losers design: normal case. *Biom J*. 2005;47(3):257-281. https://doi.org/10.1002/bimj.200410119
18. Sill MW, Sampson AR. Drop-the-losers design: binomial case. *Comput Stat Data Anal*. 2009;53(3):586-595. https://doi.org/10.1016/j.csda.2008.07.031
19. Macy ME, Kieran MW, Chi SN, et al. A pediatric trial of radiation/cetuximab followed by irinotecan/cetuximab in newly diagnosed diffuse pontine gliomas and high-grade astrocytomas: a Pediatric Oncology Experimental Therapeutics Investigators' Consortium study. *Pediatr Blood Cancer*. 2017;64(11):e26621. https://doi.org/10.1002/pbc.26621
20. Horton TM, Whitlock JA, Lu X, et al. Bortezomib reinduction chemotherapy in high-risk ALL in first relapse: a report from the Children's Oncology Group. *Br J Haematol*. 2019;186(2):15919. https://doi.org/10.1111/bjh.15919
21. Sun X, Peng P, Tu D. Phase II cancer clinical trials with a one-sample log-rank test and its corrections based on the Edgeworth expansion. *Contemp Clin Trials*. 2011;32(1):108-113.
22. Kwak M, Jung S-H. Phase II clinical trials with time-to-event endpoints: optimal two-stage designs with one-sample log-rank test. *Stat Med*. 2014;33(12):2004-2016. https://doi.org/10.1002/sim.6073
23. Wu J, Xiong X. Survival trial design and monitoring using historical controls. *Pharm Stat*. 2016;15(5):405-411. https://doi.org/10.1002/pst.1756
24. Breslow NE. Analysis of survival data under the proportional hazards model. *Int Stat Rev/Rev Int Stat*. 1975;43(1):45. https://doi.org/10.2307/1402659
25. Finkelstein DM, Muzikansky A, Schoenfeld DA. Comparing survival of a sample to that of a standard population. *J Natl Cancer Inst*. 2003;95(19):1434-1439. https://academic.oup.com/jnci/article/95/19/1434/2520464
26. Waring MJ, Arrowsmith J, Leach AR, et al. An analysis of the attrition of drug candidates from four major pharmaceutical companies. *Nat Rev Drug Discov*. 2015;14(7):475-486. https://doi.org/10.1038/nrd4609

15391612, 2022, 1, Downloaded from https://onlinelibrary.wiley.com/doi/10.1002/pst.2168 by ${individualUser.givenNames} ${individualUser.surname} - University of Cambridge , Wiley Online Library on [15/11/2022]. See the Terms and Conditions (https://onlinelibrary.wiley.com/terms-and-conditions) on Wiley Online Library for rules of use; OA articles are governed by the applicable Creative Commons License

27. Wason J, Stallard N, Bowden J, Jennison C. A multi-stage drop-the-losers design for multi-arm clinical trials. *Stat Methods Med Res*. 2017;26(1):508-524. https://doi.org/10.1177/0962280214550759
28. Genz A, Bretz F. Numerical computation of multivariate t-probabilities with application to power calculation of multiple contrasts. *J Stat Comput Simul*. 1999;63(4):361-378. https://doi.org/10.1080/00949659908811962
29. Wu J Statistical methods for survival trial design. In: *Statistical Methods for Survival Trial Design*. CRC Press; 2018:201. doi:https://doi.org/10.1201/9780429470172
30. Wu J. Sample size calculation for the one-sample log-rank test. *Pharm Stat*. 2015;14(1):26-33. https://doi.org/10.1002/pst.1654
31. Wu J. Single-arm phase II cancer survival trial designs. *J Biopharm Stat*. 2016;26(4):644-656. https://doi.org/10.1080/10543406.2015.1052494
32. Wu J. Single-arm phase II survival trial design under the proportional hazards model. *Stat Biopharm Res*. 2017;9(1):25-34. https://doi.org/10.1080/19466315.2016.1174147
33. Piperno-Neumann S, Le Deley M-C, Rédini F, et al. Zoledronate in combination with chemotherapy and surgery to treat osteosarcoma (OS2006): a randomised, multicentre, open-label, phase 3 trial. *Lancet Oncol*. 2016;17(8):1070-1080. https://doi.org/10.1016/S1470-2045(16)30096-1
34. Hommel G. A stagewise rejective multiple test procedure based on a modified bonferroni test. *Biometrika*. 1988;75:383-386. https://doi.org/10.1093/biomet/75.2.383
35. Genz A, Bretz F, Miwa T, et al. mvtnorm: multivariate normal and t distributions; 2019. http://cran.r-project.org/package=mvtnorm.
36. Royston P, Parmar MKB. Augmenting the logrank test in the design of clinical trials in which non-proportional hazards of the treatment effect may be anticipated. *BMC Med Res Methodol*. 2016;16(1):16. https://doi.org/10.1186/s12874-016-0110-x
37. Wason JMS, Brocklehurst P, Yap C. When to keep it simple – adaptive designs are not always useful. *BMC Med*. 2019;17:152. https://doi.org/10.1186/s12916-019-1391-9
38. Prentice RL. A log gamma model and its maximum likelihood estimation. *Biometrika*. 1974;61(3):539-544.
39. Stacy EW. A generalization of the gamma distribution. *Ann Math Stat*. 1962;33:1187-1192.

How to cite this article: Abbas R, Wason J, Michiels S, Le Teuff G. A two-stage drop-the-losers design for time-to-event outcome using a historical control arm. *Pharmaceutical Statistics*. 2022;21(1):268-288. doi: 10.1002/pst.2168

APPENDIX A.

The generalized gamma distribution (also known as the (generalized) log-gamma distribution) is specified using the parameterization originating from Prentice[38]: mu, sigma and Q are, respectively, the mean on the log scale, the standard deviation on the log scale and, the shape parameter.

If $\gamma \tilde{Gamma}\left(Q^{-2},1\right)$, and $w=\log\left(Q^{-2}\gamma\right)/Q$, then $x=\exp(\mu+\sigma w)$ follows the generalized gamma distribution with probability density function:

$$f(x\,|\,\mu,\sigma,Q)=\frac{|Q|\left(Q^{-2}\right)^{Q^{-2}}}{\sigma x\Gamma\left(Q^{-2}\right)}\exp\left(Q^{-2}(Qw-\exp(Qw))\right)$$

These parameters are related to the original parameters (scale, shape and k) proposed by Stacy[39] as following:

$$\mu=\log(scale)+\frac{\log(k)}{shape}$$

$$\sigma=\frac{1}{shape^{*}\sqrt{k}}$$

$$Q=\frac{1}{\sqrt{k}}$$

15391612, 2022, 1, Downloaded from https://onlinelibrary.wiley.com/doi/10.1002/pst.2168 by ${individualUser.givenNames} ${individualUser.surname} - University of Cambridge , Wiley Online Library on [15/11/2022]. See the Terms and Conditions (https://onlinelibrary.wiley.com/terms-and-conditions) on Wiley Online Library for rules of use; OA articles are governed by the applicable Creative Commons License

TABLE A1 Family-wise error rate (FWER) and empirical power over the simulated scenarios (S1–S9) of the fixed design according to total sample sizes (*N*) from 40 to 200 patients for a nominal FWER of 0.1

N	Not corrected for multiplicity[a]									Corrected with Hommel's closed test procedure								
	FWER	Power								FWER	Power							
		One effective treatment arm			Two effective treatment arms						One effective treatment arm			Two effective treatment arms				
	S1	S2	S3	S4	S5	S6	S7	S8	S9	S1	S2	S3	S4	S5	S6	S7	S8	S9
40	0.29	0.27	0.45	0.62	0.46	0.60	0.70	0.79	0.86	0.12	0.14	0.27	0.42	0.25	0.37	0.47	0.59	0.68
60	0.27	0.32	0.55	0.75	0.53	0.69	0.80	0.89	0.94	0.11	0.17	0.36	0.57	0.30	0.47	0.60	0.73	0.82
80	0.26	0.35	0.63	0.83	0.57	0.75	0.86	0.94	0.97	0.10	0.19	0.43	0.67	0.33	0.54	0.68	0.82	0.90
100	0.25	0.38	0.69	0.89	0.62	0.82	0.90	0.97	0.99	0.09	0.21	0.50	0.77	0.37	0.61	0.76	0.89	0.95
120	0.24	0.42	0.76	0.93	0.66	0.86	0.94	0.98	1.00	0.09	0.25	0.58	0.84	0.42	0.68	0.82	0.94	0.98
140	0.25	0.44	0.79	0.95	0.70	0.89	0.96	0.99	1.00	0.09	0.26	0.62	0.88	0.46	0.73	0.87	0.96	0.99
160	0.24	0.49	0.84	0.97	0.73	0.91	0.97	1.00	1.00	0.09	0.29	0.69	0.93	0.50	0.78	0.91	0.98	0.99
200	0.22	0.53	0.89	0.99	0.78	0.95	0.99	1.00	1.00	0.08	0.34	0.77	0.96	0.57	0.86	0.95	0.99	1.00

[a]Three single arm trials considered as three independent trials.

15391612, 2022, 1, Downloaded from https://onlinelibrary.wiley.com/doi/10.1002/pst.2168 by ${individualUser.givenNames} ${individualUser.surname} - University of Cambridge , Wiley Online Library on [15/11/2022]. See the Terms and Conditions (https://onlinelibrary.wiley.com/terms-and-conditions) on Wiley Online Library for rules of use; OA articles are governed by the applicable Creative Commons License

TABLE A2 Family-wise error rate (FWER) and empirical power over the simulated scenarios (S1–S9) of the fixed design according to total sample sizes (*N*) from 40 to 200 patients for a nominal FWER of 0.05

N	Not corrected for multiplicity[a]									Corrected with Hommel's closed test procedure								
		Power									Power							
	FWER	One effective treatment arm				Two effective treatment arms				FWER	One effective treatment arm			Two effective treatment arms				
	S1	S2	S3	S4	S5	S6	S7	S8	S9	S1	S2	S3	S4	S5	S6	S7	S8	S9
40	0.16	0.17	0.32	0.48	0.31	0.43	0.54	0.65	0.73	0.06	0.09	0.18	0.31	0.16	0.25	0.34	0.45	0.54
60	0.15	0.20	0.42	0.63	0.37	0.54	0.66	0.79	0.86	0.06	0.10	0.26	0.45	0.20	0.34	0.45	0.60	0.71
80	0.14	0.23	0.49	0.73	0.40	0.60	0.74	0.86	0.93	0.05	0.12	0.32	0.57	0.22	0.40	0.53	0.71	0.82
100	0.13	0.25	0.56	0.81	0.45	0.67	0.81	0.92	0.96	0.05	0.14	0.39	0.67	0.25	0.47	0.64	0.81	0.90
120	0.13	0.29	0.63	0.88	0.49	0.74	0.87	0.96	0.98	0.05	0.16	0.46	0.76	0.30	0.55	0.71	0.87	0.94
140	0.13	0.32	0.68	0.91	0.54	0.78	0.91	0.97	0.99	0.05	0.17	0.51	0.81	0.32	0.60	0.78	0.92	0.97
160	0.12	0.35	0.74	0.95	0.57	0.83	0.93	0.98	1.00	0.05	0.20	0.58	0.87	0.36	0.67	0.83	0.95	0.99
200	0.11	0.40	0.82	0.97	0.64	0.89	0.97	1.00	1.00	0.04	0.24	0.67	0.93	0.43	0.76	0.90	0.98	1.00

[a]Three single arm trials considered as three independent trials.

15391612, 2022, 1, Downloaded from https://onlinelibrary.wiley.com/doi/10.1002/pst.2168 by ${individualUser.givenNames} ${individualUser.surname} - University of Cambridge , Wiley Online Library on [15/11/2022]. See the Terms and Conditions (https://onlinelibrary.wiley.com/terms-and-conditions) on Wiley Online Library for rules of use; OA articles are governed by the applicable Creative Commons License
